# Prevalence and predictors of small intestinal bacterial overgrowth in inflammatory bowel disease: a meta-analysis

**DOI:** 10.3389/fmed.2024.1490506

**Published:** 2025-01-21

**Authors:** Xin Feng, Jie Hu, Xin Zhang

**Affiliations:** ^1^Department of Gastroenterology, The People’s Hospital of Yubei District of Chongqing City, Chongqing, China; ^2^Department of Ultrasound, The People’s Hospital of Yubei District of Chongqing City, Chongqing, China

**Keywords:** small intestinal bacterial overgrowth, inflammatory bowel disease, meta-analysis, ulcerative colitis, Crohn’s disease

## Abstract

**Background:**

Small intestinal bacterial overgrowth (SIBO) has been reported to be very common among individuals with inflammatory bowel disease (IBD), and the prevalence of SIBO is highly variable. We conducted this study to calculate the prevalence and identify predictors of SIBO in IBD.

**Methods:**

PubMed, Cochrane Library, and EMBASE from inception to March 2024 were searched for studies evaluating the prevalence of SIBO in IBD. We calculated the pooled prevalence of SIBO among IBD patients and the odds ratio (OR) of SIBO in IBD compared with healthy controls. Besides, we also evaluated predictors of SIBO in IBD patients.

**Results:**

Twenty-nine studies (3,250 IBD, 708 controls) were included in our study. The pooled prevalence of SIBO in IBD was 31.0% (95% CI 25.2–37.1), and the prevalence of SIBO was higher in IBD compared with healthy controls (OR 5.25, 95% CI 2.96–9.32). The pooled prevalence of SIBO was higher among CD patients (32.2, 95% CI 25.9–38.8) compared with UC patients (27.8, 95% CI 18.5–38.1). The odds of lower BMI (mean difference = −1.04; 95% CI −1.86 to −0.23), bloating (OR = 3.02, 95% CI 1.22–7.5), flatulence (OR = 4.70, 95% CI 1.44–15.35), history of abdominal surgery (OR = 2.05, 95% CI 1.35–3.11), and stricturing/penetrating disease behavior (OR = 3.51, 95% CI 1.67–7.40) increased significantly in IBD patients with SIBO compared to those without SIBO. Antibiotic treatment may be effective for SIBO in IBD patients.

**Conclusion:**

Nearly one-third of IBD patients present with SIBO positive, and the odds of SIBO in IBD was increased by 5.25-fold compared with healthy controls. Lower BMI, bloating, flatulence, history of abdominal surgery, and stricturing/penetrating disease behavior were predictors of SIBO in IBD patients.

## Introduction

Inflammatory bowel disease (IBD) is a chronic or remitting/relapsing inflammatory disease of the gastrointestinal tract characterized by abdominal pain, diarrhea, bloody stools, and weight loss, including ulcerative colitis (UC) and Crohn’s disease (CD) (1, 2). Over the past few decades, the prevalence of IBD has been increasing around the world (1, 3, 4). The recurrent symptoms of IBD require frequent medical evaluation and treatments, resulting in a substantial economic and psychological burden. Although the pathogenesis of IBD remains unclear, the abnormalities in disease susceptibility genes, environmental factors, and intestinal bacteria are associated with the development or progression of IBD (1).

Small intestinal bacterial overgrowth (SIBO) is a clinical syndrome caused by excessive numbers of bacteria and/or abnormal types of bacteria in the small intestinal tract (5, 6). The microbial investigation of jejunal aspirate culture (JAC) has been the gold standard for diagnosing SIBO. However, due to the invasiveness, expensiveness, and complexity of JAC, the breath test has become the mainstream method for diagnosing SIBO in real clinical practice (7). Many clinical symptoms of SIBO, such as abdominal pain, diarrhea, and weight loss, are similar to those of IBD. Hence, gastrointestinal (GI) symptoms seen in SIBO may be confused with IBD symptoms. The latest guidelines have suggested that dysbiosis of intestinal flora is also an important pathogenesis of IBD (1). Regrettably, the optimal role of SIBO in the development of IBD has not been established.

In this context, there is much interest in the possible association between SIBO and IBD. A series of clinical trials have assessed the prevalence of SIBO in IBD patients, but the reported result is highly variable, ranging from 9 to 62%. A previous meta-analysis published in 2019 concluded that the proportion of SIBO in IBD patients was 22.3% (8). However, the number of studies included in this research was very limited, and they did not quantitatively analyze the predictors of SIBO in IBD patients. Another meta-analysis published in 2021 reported that the prevalence of methane-positive SIBO in IBD patients was 5.6% (9). However, this meta-analysis did not include hydrogen-positive SIBO patients, which led to the result not truly demonstrating the condition of SIBO in IBD patients. Recognizing that previous studies might not be able to provide convincing data to affect practice, we conducted an updated meta-analysis.

The primary aim of our study was to evaluate the pooled prevalence rates of SIBO among individuals with IBD, the pooled odds ratio (OR) of SIBO among IBD patients compared with controls, and also to examine predictors associated with SIBO among IBD.

## Methods

### Search strategy and selection criteria

This meta-analysis is performed with the Preferred Reporting Items for Systematic Reviews and Meta-Analyses (PRISMA) Statement and was registered at the International Prospective Register of Systematic Reviews (PROSPERO ID: CRD42024521031) (10).

We selected relevant studies published from inception to June 2024 by searching PubMed, Embase, and Cochrane Library. We applied no language restrictions. We used the following search strategy: [‘SIBO’ OR ‘small intestinal bacterial overgrowth’ OR ‘small intestine bacterial overgrowth’ OR ‘breath test’ OR ‘small bowel bacterial overgrowth’ OR ‘SBBO’] AND [‘IBD’ OR ‘inflammatory bowel disease’ OR ‘UC’ OR ‘Ulcerative colitis’ OR ‘CD’ OR ‘Crohn’s disease’]. We manually searched the reference lists of all the included articles to help identify additional potentially relevant studies. We tried to contact the authors if we could not get a full article.

### Study selection and data extraction

The inclusion criteria were: (1) case-series study or case–control study; (2) studies recruiting subjects meeting diagnostic criteria for IBD, including clinical, radiological, colonoscopy, and histological diagnosis. (3) studies of SIBO being diagnosed using valid methods (breath test or JAC). (4) studies that reported the prevalence of SIBO in IBD patients or compared the prevalence of SIBO in IBD patients versus healthy controls. (5) studies included more than 40 individuals. The exclusion criteria were: (1) case reports, review articles, letters, and animal studies; (2) studies with inaccurate data. Two independent investigators (X Feng and J Hu) searched and assessed study titles, abstracts, and full-text.

Two investigators (X Feng and J Hu) extracted the following data from each selected study: first author, the year of study, study design, country, age, gender, sample sizes of IBD (UC, CD), and controls, diagnostic criteria for IBD, source of controls, a diagnostic test of SIBO including dose of substrate, cut off criteria for positive SIBO diagnosis and test duration, the proportion of SIBO in IBD (UC and CD) patients and controls, prior antibiotic use, concurrent PPI use, history of abdominal surgery, antibiotics treatment of SIBO positive patients and the improvements of main symptoms post-treatment.

The quality of the included case–control studies was assessed using the Newcastle-Ottawa scale (NOS) based on the following three domains: the selection of subjects, the comparability of groups, and the ascertainment of exposure of interest (11). The quality of the study was ranked as high when the study reached the score of 7 stars, moderate when the study reached the score of 4–6 stars, and low when the study was below the score of 4 stars. In addition, the quality of the included case-series studies was assessed using the Joanna Briggs Institute (JBI) Critical Appraisal Tools (12). Studies with scores ≥7 “yes” were considered low risk of bias, scores of 5 “yes” or 6 “yes” were considered moderate risk of bias, and scores <5 “yes” were considered high risk of bias. A third investigator (X Zhang) resolved disagreements between the two investigators.

### Statistical analysis

We estimated the pooled prevalence of SIBO in all individuals with IBD. For case–control studies, we calculated the pooled odds ratio (OR) and 95% confidence interval (CI) by comparing the prevalence of positive SIBO between the IBD group and the control group. Further, meta-analyses, according to predictors, such as demographic (gender, age), abdominal symptoms (abdominal pain, bloating), and history of abdominal operation, were also estimated.

A *p* value <0.05 was considered statistically significant. We used the Cochran *Q* test and *I*^2^ testing to assess heterogeneity between studies, with chi-squared test *p* < 0.10 or *I*^2^ ≥ 50% regarded as substantial heterogeneity (13, 14). We used the fixed-effects model in low heterogeneity (*I*^2^ < 50%) and the random-effects model in high heterogeneity (*I*^2^ ≥ 50%). We further conducted subgroup analyses stratified by diagnostic methods or quality of the studies to analyze the sources of heterogeneity among pooled studies. Furthermore, we constructed a funnel plot to assess the possibility of publication bias. We performed the Egger test to evaluate funnel plot asymmetry, with a *p*-value <0.05 indicating significant publication bias. We used RevMan 5.3 and Stata 12.0 for all statistical analyses.

## Results

### Study selection

**Figure 1:**
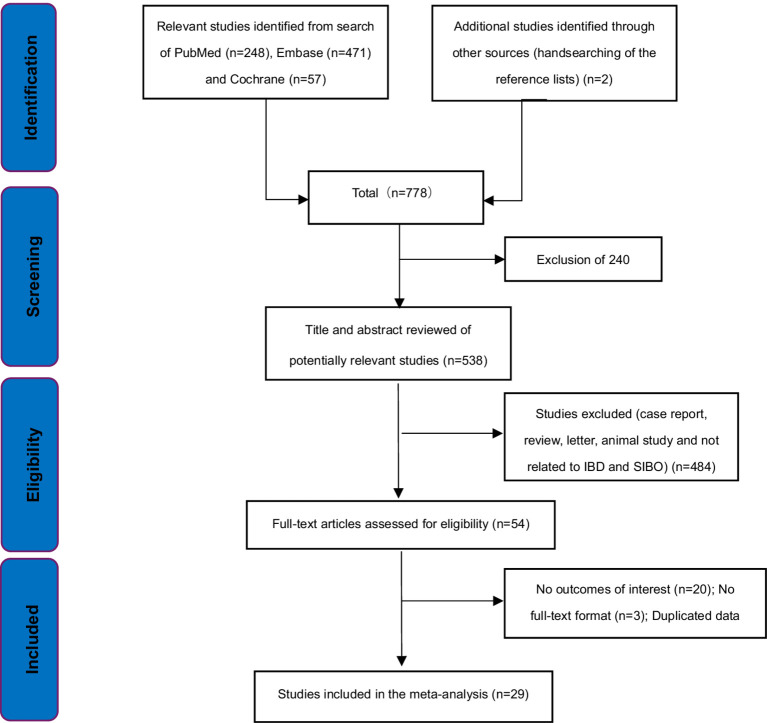
Flow chart of the selection process of articles.

We identified 778 potentially relevant articles based on the search strategy. 240 articles were excluded for duplicates. 509 articles did not meet the inclusion criteria and were excluded after evaluation on the title/abstract/full-text level. Finally, 29 studies were included in our analysis (15–43) (Figure 1). 18 of the 29 studies were case-series studies (15–18, 24–27, 29, 31, 33, 34, 37–40, 42, 43) and the remaining 11 were case–control studies (19–23, 28, 30, 32, 35, 36, 41). Ten case–control studies included healthy volunteers as controls, and only one study included individuals undergoing nonspecific, nonchronic (duration <3 months) GI symptoms as controls (28). Four of these studies included UC patients (20, 22, 24, 35), 13 included CD patients (17, 18, 26–28, 30, 33, 34, 37–39, 41, 43), while 12 included both UC and CD patients (15, 16, 19, 21, 23, 25, 29, 31, 32, 36, 40, 42). In addition, 12 of the 18 case-series studies were considered low risk of bias (15–18, 25, 26, 29, 31, 33, 34, 37, 39), and six were considered moderate risk of bias (24, 27, 38, 40, 42, 43). Nine of the 11 case–control studies were ranked high quality (19–23, 28, 32, 35, 36), and two were considered moderate quality (30, 41). Twenty studies excluded patients who had previously received antibiotic treatment (15–17, 20–23, 26, 28, 31–37, 39–41, 43). Lastly, only one study was available in Russian (16), and the other 28 were in English. The main characteristics and quality evaluation of all the studies included in this study are shown in Table 1 and Supplementary Tables S1, S2.

**Table 1:** Main characteristics of the studies included in this meta-analysis.

No	Author	Study year	Study type	Country	Mean age (Years)	Male (%)	IBD, n	UC, n	CD, n	Diagnostic criteria for IBD	Controls, n	Type of control	Diagnostic test of SIBO	SIBO in IBD patients, *n* (%)	SIBO in UC patients, *n* (%)	SIBO in CD patients, *n* (%)	SIBO in controls, *n* (%)
1	Wanzl J (15)	2023	Case-series study	Germany	58.9 ± 18.7	42.2	74	22	52	Known IBD	NA	NA	GBT	13 (17.6)	2 (9.1)	11 (21.2)	NA
2	Kulygin Y (16)	2023	Case-series study	Russia	NA	NA	152	81	71	*IBD diagnosis	NA	NA	LBT	73 (48)	37 (45.7)	36 (50.7)	NA
3	Rajan A (17)	2023	Case-series study	USA	41 ± 16	26	219	NA	219	Known IBD	NA	NA	GBT (*n* = 2) LBT (*n* = 217)	114 (52)	NA	114 (52)	NA
4	Wei J (18)	2022	Case-series study	China	26.0 (20–41)	57.5	73	NA	73	Known IBD	NA	NA	LBT	34 (46.6)	NA	34 (46.6)	NA
5	Ghoshal UC (19)	2022	Case–control study	India	38.2 ± 12.1 (IBD) 41.2 ± 12.8 (controls)	62.8 (IBD) 71.2 (controls)	86	45	41	*IBD diagnosis	66	Healthy controls	GBT	16 (18.6)	2 (4.4)	14 (34.1)	1 (1.5)
6	Yang C (20)	2021	Case–control study	China	42.9 ± 4.3 (IBD) 41.7 ± 4.4 (controls)	56 (IBD) 52.5 (controls)	89	89	NA	Known IBD	40	Healthy controls	LBT	50 (56.2)	50 (56.2)	NA	10 (25)
7	Tong Y (21)	2021	Case–control study	China	52.5 ± 15.9 (UC) 46.7 ± 13.9 (CD) 48.6 ± 14.2 (controls)	51 (UC) 56 (CD) 53 (controls)	71	39	32	Known IBD	30	Healthy controls	LBT	24 (33.8)	12 (30.8)	12 (37.5)	2 (6.7)
8	Yang C (22)	2020	Case–control study	China	46.3 ± 3.2 (IBD) 45.8 ± 2.7 (controls)	53.9 (IBD) 52.8 (controls)	130	130	NA	*IBD diagnosis	72	Healthy controls	LBT	57 (43.8)	57 (43.8)	NA	9 (12.5)
9	Shah A (23)	2020	Case–control study	Australia	44.2 ± 13.4 (IBD) 57.0 ± 13.2 (controls)	46.7 (IBD) 55.6 (controls)	81	48	33	*IBD diagnosis	44	Healthy controls	GBT	12 (14.8)	9 (18.8)	3 (9.1)	8 (18,1)
10	Lorio EA (24)	2020	Case-series study	USA	55.6 ± 18.8	49.2	67	67	NA	Known IBD	NA	NA	LBT	21 (31.3)	21 (31.3)	NA	NA
11	Gu P (25)	2020	Case-series study	USA	42.3 ± 16.1 (UC) 40.8 ± 15.5 (CD)	40 (UC) 40 (CD)	465	175	290	Known IBD	NA	NA	LBT	264 (56.8)	101 (57.7)	163 (56.2)	NA
12	Bertges ER (26)	2020	Case-series study	Brazil	37.1 ± 19.1	40	110	NA	110	*IBD diagnosis	NA	NA	GBT	33 (30)	NA	33 (30)	NA
13	Kulygina Y (27)	2019	Case-series study	Russia	NA	NA	71	NA	71	Known IBD	NA	NA	LBT	36 (51)	NA	36 (51)	NA
14	Ricci J (28)	2018	Case–control study	Brazil	39.7 ± 12.5 (CD) 37.6 ± 14.2 (controls)	37 (CD) 41 (controls)	92	NA	92	*IBD diagnosis	97	Nonspecific, nonchronic GI symptoms	GBT	30 (32.6)	NA	30 (32.6)	12 (12.4)
15	Cohen-Mekelburg S (29)	2018	Case-series study	USA	40.9 ± 14.4	34	147	74	73	Known IBD	NA	NA	LBT	91 (61.9)	46 (50.6)	45 (49.4)	NA
16	Chen L (30)	2018	Case–control study	China	NA	NA	50	NA	50	Known IBD	49	Healthy controls	LBT	23 (46)	NA	23 (46)	5 (10.2)
17	Andrei M (31)	2016	Case-series study	Romania	47.3 ± 14.1	49.3	75	36	39	Known IBD	NA	NA	GBT	19 (25.3)	7 (19.4)	12 (30.8)	NA
18	Lee J (32)	2015	Case–control study	Korea	41.8 ± 15.0 (IBD) 40.3 ± 16.1 (controls)	64.5 (IBD) 43.3 (controls)	107	64	43	Known IBD	30	Healthy controls	GBT	22 (20.6)	9 (14.1)	13 (30.2)	2 (6.7)
19	Greco A (33)	2015	Case-series study	Italy	49.3 ± 12.8	61.8	68	NA	68	*IBD diagnosis	NA	NA	GBT	18 (26.5)	NA	18 (26.5)	NA
20	Sánchez-Montes C (34)	2014	Case-series study	Spain	40.8 ± 12.1	47.7	107	NA	107	Known IBD	NA	NA	GBT	18 (16.8)	NA	18 (16.8)	NA
21	Rana S (35)	2014	Case–control study	India	45.6 ± 17.5 (UC) 44.7 ± 19.5 (controls)	61.7 (UC) 58.4 (controls)	120	120	NA	*IBD diagnosis	125	Healthy controls	GBT	18 (15)	18 (15)	NA	2 (1.6)
22	Rana S (36)	2013	Case–control study	India	42.7 ± 19.3 (UC) 44.5 ± 18.6 (CD) 45.3 ± 21.6 (controls)	64.2 (UC) 64.3 (CD) 60.8 (controls)	137	95	42	Colonic biopsy	115	Healthy controls	GBT	36 (26.3)	17 (17.8)	19 (45.2)	1 (0.86)
23	Klaus J (37)	2009	Case-series study	Germany	41 (18–72)	39.3	150	NA	150	*IBD diagnosis	NA	NA	GBT	38 (25.3)	NA	38 (25.3)	NA
24	Tursi A (38)	2003	Case-series study	Italy	41.6 (22–73)	64.4	45	NA	45	*IBD diagnosis	NA	NA	LBT	4 (8.9)	NA	4 (8.9)	NA
25	Castiglione F (39)	2003	Case-series study	Italy	38.6 (21–70)	54.5	145	NA	145	Known IBD	NA	NA	GBT	29 (20)	NA	29 (20)	NA
26	Mishkin D (40)	2002	Case-series study	Canada	49 (GBT+) 41 (GBT-)	50.7	117	46	71	Known IBD	NA	NA	GBT	27 (23.1)	3 (6.5)	24 (33.8)	NA
27	Castiglione F (41)	2000	Case–control study	Italy	39.5 ± 16 (CD) 34 ± 12 (controls)	65.7 (CD) 58.1 (controls)	57	NA	57	Known IBD	40	Healthy controls	LBT	13 (22.8)	NA	13 (22.8)	0 (0)
28	Peled Y (42)	1987	Case-series study	Israel	47 (16–84) (IBD)	48.3 (IBD)	84	51	33	*IBD diagnosis	NA	NA	methane breath test	18 (21.4)	16 (31.4)	2 (6.1)	NA
29	Rutgeerts P (43)	1981	Case-series study	Belgium	17–58	49.2	61	NA	61	Known IBD	NA	NA	14C-glycocholate breath test	15 (25)	NA	15 (25)	NA

IBD, inflammatory bowel disease; UC, ulcerative colitis; CD, Crohn’s disease; SIBO, small intestinal bacterial overgrowth; LBT, lactulose breath test; GBT, glucose breath test; NA, not available; GI, gastrointestinal; *IBD diagnosis, clinical, radiological, colonoscopy and histological diagnosis of IBD.

### Study population and testing for SIBO

A total of 3,250 individuals with IBD (1,182 UC and 2,068 CD) and 708 healthy controls were included in the 29 studies. These together comprised 12 studies from Asia (16, 18–22, 27, 30, 32, 35, 36, 42), 9 from Europe (15, 31, 33, 34, 37–39, 41, 43), 7 from the Americas (17, 24–26, 28, 29, 40), 1 from Australia (23). SIBO was defined by using GBT in 14 (15, 19, 23, 26, 28, 31–37, 39, 40), LBT in 13 (16–18, 20–22, 24, 25, 27, 29, 30, 38, 41), methane breath test in one (42), and 14C-glycocholate breath test in one (43) (Table 1). Of the 14 studies that used GBT to diagnose SIBO, the substrate dose was 50 g glucose in seven studies, 75 g glucose in four, 80 g glucose in two, and 100 g glucose in one. Of the 13 studies that used LBT to diagnose SIBO, the substrate dose was 10 g lactulose in nine studies, and four studies did not specify the substrate dose. Multiple cut-off criteria to define a positive SIBO diagnosis were observed in the included studies. In the 14 studies that used GBT to measure hydrogen or methane production, positive SIBO was diagnosed by a rise of >12 parts per million (ppm) above baseline in 10 studies, a rise of >10 ppm above baseline in two studies, a rise of >20 ppm above baseline in two studies. In the 13 studies that used LBT to measure hydrogen or methane production, positive SIBO was diagnosed by a rise of >20 ppm above baseline in five studies, baseline >20 ppm in one study, a rise of >12 ppm above baseline and/or baseline >20 ppm in two studies, a rise of >10 ppm above baseline and/or baseline >20 ppm in one study, a rise of >12 ppm above baseline in one study, a rise of >10 ppm above baseline in one study, and criteria not specified in two studies. In the study that used methane breath test, positive SIBO was diagnosed when the methane gas level was at least 1 ppm above ambient air (42). The study that used 14C-glycocholate breath test did not specify criteria (43). The cut off criteria for diagnosing SIBO in IBD patients are included in Supplementary Table S3.

### Prevalence of SIBO in IBD patients

**Figure 2:**
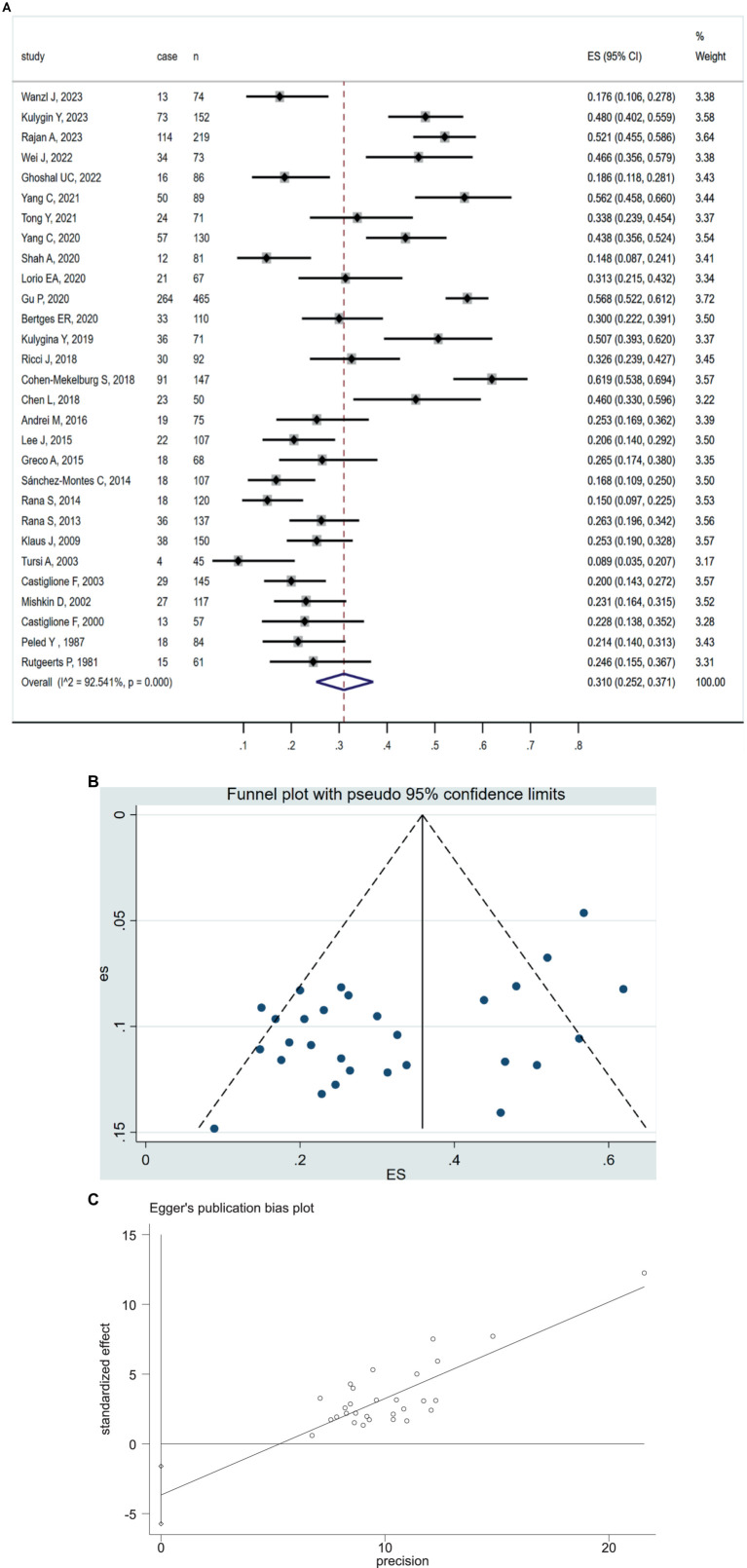
**(A)** Forest plot of studies showing pooled prevalence of SIBO in patients with IBD (31.0% [95% CI 25.2–37.1]), (*I*^2^ = 92.54, *p* = 0.0001). **(B)** Funnel plot of positive SIBO in patients with IBD. **(C)** Egger’s publication bias plot of positive SIBO in patients with IBD (*p* = 0.001).

All 29 included studies reported the prevalence of positive SIBO in IBD patients. The pooled prevalence of SIBO in IBD population was 31.0% (95% CI 25.2–37.1) (Figure 2A). The highest prevalence of SIBO in IBD was 61.9% (29), and the lowest prevalence was 8.9% (38). Given the significantly high heterogeneity (*I*^2^ = 92.5%, *p* < 0.0001) detected among the included studies, we applied a random-effects model. The asymmetry of the funnel plot revealed the presence of publication bias (Figure 2B), which was further confirmed by the result of Egger’s test (*p* = 0.001) (Figure 2C). To explore the variability of the prevalence of SIBO among the studies, we conducted subgroup analyses based on IBD subtypes, SIBO diagnostic tests, quality of study, geographic area. The prevalence of SIBO in CD patients (32.2, 95% CI 25.9–38.8) was higher than in UC patients (27.8, 95% CI 18.5–38.1) (Supplementary Figure S1). The subgroup analysis also showed that the prevalence of SIBO was higher in studies using the LBT (43.3, 95% CI 36.2–50.6) than those using the GBT (22.1, 95% CI 19.3–25.0) or other breath tests (22.7, 95% CI 16.2–30.0) (Supplementary Figure S2). Furthermore, in subgroup analysis by quality of study, the prevalence of SIBO was 32.2% (95% CI 25.2–39.6) in studies with high quality or low risk of bias and 27.8% (95% CI 19.4–37.1) in studies with moderate quality or moderate risk of bias (Supplementary Figure S3). Finally, the prevalence of SIBO in Eastern countries (34.7, 95% CI 26.4–43.5) was greater than the prevalence in Western countries (28.4, 95% CI 20.5–37.0) (Supplementary Figure S4). The summary of the subgroup analyses is included in Table 2.

**Table 2:** The summary of the results of the subgroup analyses evaluating prevalence of SIBO in IBD.

Subgroups	Number of studies	Prevalence of SIBO (%) (95% CI)	Heterogeneity
IBD subtypes			
UC	16 (1,182 UC patients)	27.8 (18.5–38.1)	*I*^2^ = 92.8%, *p* = 0.0001
CD	25 (2,068 CD patients)	32.2 (25.9–38.8)	*I*^2^ = 89.5%, *p* = 0.0001
SIBO diagnostic tests			
LBT	13 (1,636 IBD patients)	43.3 (36.2–50.6)	*I*^2^ = 87.5%, *p* = 0.0001
GBT	14 (1,469 IBD patients)	22.1 (19.3–25.0)	*I*^2^ = 0%, *p* = 0.42
Other breath tests	2 (145 IBD patients)	22.7 (16.2–30.0)	–
Quality of study			
high quality/low risk of bias	21 (2,698 IBD patients)	32.2 (25.2–39.6)	*I*^2^ = 93.8%, *p* = 0.0001
moderate quality/moderate risk of bias	8 (552 IBD patients)	27.8 (19.4–37.1)	*I*^2^ = 81.5%, *p* = 0.0001
Geographic areas			
Eastern countries	12 (1,170 IBD patients)	34.7 (26.4–43.5)	*I*^2^ = 89.6%, *p* = 0.0001
Western countries	17 (2,080 IBD patients)	28.4 (20.5–37.0)	*I*^2^ = 94.1%, *p* = 0.0001

IBD, Inflammatory Bowel Disease; UC, ulcerative colitis; CD, Crohn’s disease; LBT, Lactulose breath test; GBT, Glucose breath test; CI, confidence interval.

### Prevalence of SIBO in IBD patients and controls

**Figure 3:**
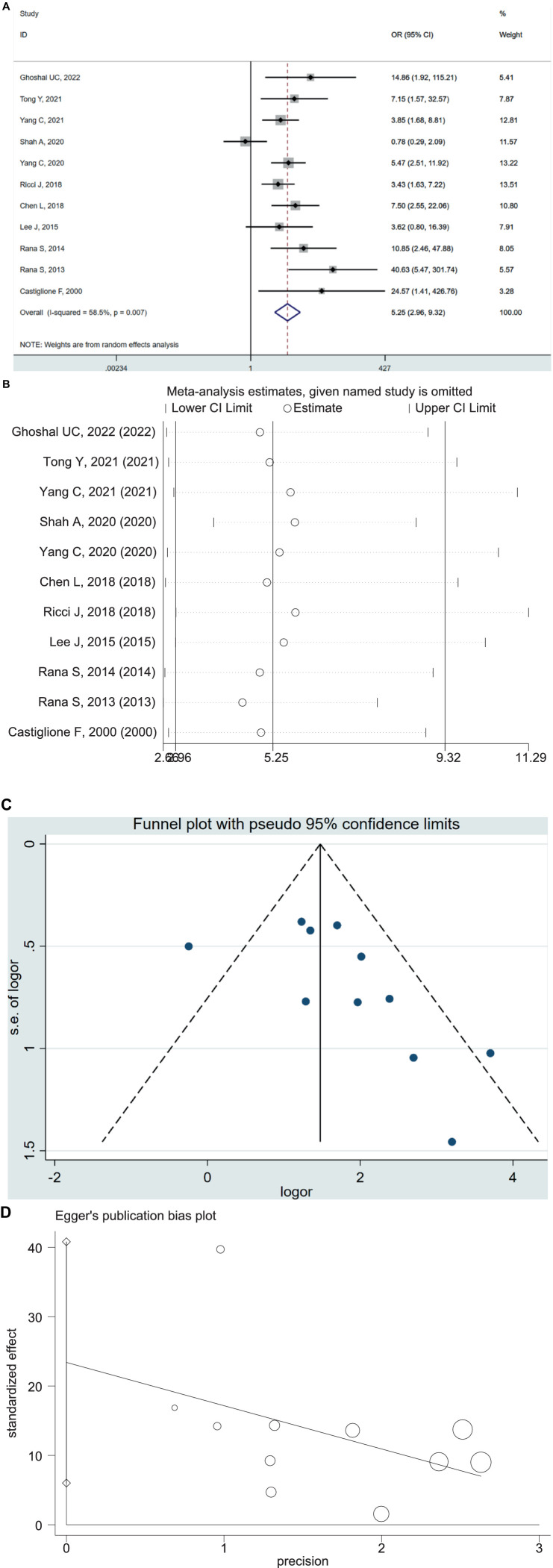
**(A)** Forest plot of odds ratios of SIBO in IBD patients compared with healthy controls (OR = 5.25 [95% CI 2.96–9.32]), (*I*^2^ = 58.5, *p* = 0.007). **(B)** Sensitivity analysis plot of odds ratios of SIBO in IBD patients compared with healthy controls. **(C)** Funnel plot showing the publication bias of odds ratios of SIBO in IBD compared with healthy controls. **(D)** Egger’s publication bias plot of odds ratios of SIBO in IBD compared with healthy controls (*p* = 0.014).

The 11 case–control studies included 1,020 patients with IBD (630 UC and 390 CD) and 708 controls. The pooled OR of SIBO in IBD patients compared with healthy controls was 5.25 (95% CI 2.96–9.32, *p* < 0.00001) (Figure 3A), with moderate heterogeneity detected among the studies (*I*^2^ = 59%, *p* = 0.007). We performed a sensitivity analysis, which indicated that no single research was biasing the results (Figure 3B). The visual inspection of the funnel plot showed that no significant publication bias existed (Figure 3C), which is consistent with the result of Egger’s test (*p* = 0.014) (Figure 3D). Similarly, the subgroup analysis based on IBD subtype concluded the increased prevalence of SIBO in UC patients (OR = 4.22; 95% CI 2.26–7.85) and CD patients (OR = 7.34; 95% CI 2.69–20.05) (Supplementary Figures S5, S6).

### Predictors of SIBO in patients with IBD

We conducted quantitative analyses to examine demographic and clinical factors that potentially impact the prevalence of SIBO in the IBD population. Four studies (*N* = 384 IBD patients) assessed the difference in BMI between patients with and without SIBO. Pooling the data of these studies demonstrated that IBD patients with SIBO had a lower BMI than those without SIBO (mean difference (MD) = −1.04; 95% CI −1.86 to −0.23), which was statistically significant (*p* = 0.01) (Supplementary Figure S7). Eight studies (*N* = 897 IBD patients) assessed the OR of bloating between patients with and without SIBO. Pooling the data of these studies showed that the prevalence of bloating was greater in patients with SIBO than those without SIBO (OR = 3.02, 95% CI 1.22–7.51; *p* = 0.02) (Supplementary Figure S8). Similarly, the OR of flatulence was also assessed by six studies (*N* = 679 IBD patients). The result showed that IBD patients with SIBO had a greater prevalence of flatulence than those without SIBO (OR = 4.70, 95% CI 1.44–15.35; *p* = 0.01) (Supplementary Figure S9). Pooled analysis of 14 studies (*N* = 1,822 IBD patients) showed a higher likelihood of a history of abdominal surgery in IBD patients with SIBO compared to those without SIBO (OR = 2.05, 95% CI 1.35–3.11; *p* = 0.0007) (Supplementary Figure S10). Pooled analysis of 11 studies (*N* = 1,220 IBD patients) that assessed the CD behavior (B1: Inflammatory; B2: Structuring; B3: Penetrating) showed that CD patients with SIBO had a higher likelihood of B2 or B3 behavior than those without SIBO (OR = 3.51, 95% CI 1.67–7.40; *p* = 0.0009) (Supplementary Figure S11). Furthermore, other meta-analyses demonstrated that the differences in the mean age, gender, disease duration, disease location (L1: ileal; L2: colonic; L3: ileocolonic), abdominal pain, diarrhea, steroids, immunomodulator, and smoking were not statistically significant between IBD patients with and without SIBO (Supplementary Figures S12–S20). The summary of the meta-analyses for the predictors of SIBO in IBD is included in Table 3.

**Table 3:** The summary of the meta-analyses for the predictors of SIBO in patients with IBD.

Predictors	Number of studies	OR/MD (95% CI)	Heterogeneity
BMI	4 (384 IBD patients)	MD = −1.04 (−1.86 to −0.23); *p* = 0.01	*I*^2^ = 0%, *p* = 0.52
Bloating	8 (897 IBD patients)	OR = 3.02 (1.22–7.51); *p* = 0.02	*I*^2^ = 86%, *p* < 0.00001
Flatulence	6 (679 IBD patients)	OR = 4.70 (1.44–15.35); *p* = 0.01	*I*^2^ = 89%, *p* < 0.00001
History of abdominal surgery	14 (1,822 IBD patients)	OR = 2.05 (1.35–3.11); *p* = 0.0007	*I*^2^ = 63%, *p* = 0.0008
CD behavior	11 (1,220 IBD patients)	OR = 3.51 (1.67–7.40); *p* = 0.0009	*I*^2^ = 84%, *p* < 0.00001
Disease duration	5 (452 IBD patients)	MD = 0.40 (−0.08 to 0.88); *p* = 0.10	*I*^2^ = 0%, *p* = 0.42
CD location	6 (779 IBD patients)	OR = 1.06 (0.79–1.43); *p* = 0.69	*I*^2^ = 0%, *p* = 0.77
Abdominal pain	8 (897 IBD patients)	OR = 1.48 (0.97–2.27); *p* = 0.07	*I*^2^ = 42%, *p* = 0.10
Diarrhea	5 (651 IBD patients)	OR = 1.33 (0.70–2.50); *p* = 0.38	*I*^2^ = 62%, *p* = 0.03
Steroids	4 (494 IBD patients)	OR = 0.75 (0.44–1.28); *p* = 0.30	*I*^2^ = 0%, *p* = 0.62
Immunomodulator	5 (601 IBD patients)	OR = 1.10 (0.73–1.65); *p* = 0.65	*I*^2^ = 0%, *p* = 0.88
Age	6 (743 IBD patients)	OR = 0.90 (−1.88 to 3.69); *p* = 0.53	*I*^2^ = 29%, *p* = 0.22
Smoking	5 (955 IBD patients)	OR = 1.23 (0.77–1.96); *p* = 0.38	*I*^2^ = 0%, *p* = 0.99
Male	8 (866 IBD patients)	OR = 0.82 (0.57–1.18); *p* = 0.29	*I*^2^ = 27%, *p* = 0.21

OR, odds ratio; MD, mean difference; CI, confidence interval; A *p* value < 0.05 was considered statistically significant.

### Effect of antibiotic treatment on IBD patients with SIBO

Six studies evaluated the effect of antibiotic treatment in IBD patients with SIBO (20, 25, 29, 33, 38, 39) (Supplementary Table S4). Yang et al. (20) divided 50 UC patients into group A (mesalazine) and group B (mesalazine + rifaximin) to compare the clinical efficacy. They found that group B presented a greater total effective rate than group A (92.86% vs. 63.64%, *p* < 0.05) and led to a greater reduction in the level of ESR and CRP (all *p* < 0.05). Gu et al. (25) treated 117 IBD patients with antibiotics for 2 weeks, and 57.3% of patients showed symptomatic improvement significantly. Cohen-Mekelburg et al. (29) observed a significant reduction in both the median Mayo Score and the median HBI score after the treatment with rifaximin and probiotics. Greco et al. (33) treated 15 CD patients with various antibiotic treatments (ciprofloxacin, metronidazole, or rifaximin). After treatment, normalization of GBT in 13/15 patients and a significant increase in vitamin B12 levels (*p* = 0.011) were reported. Similarly, Castiglione (39) reported normalization of GBT in 27/29 CD patients and significant improvement in GI symptoms after antibiotic treatment (metronidazole or ciprofloxacin). Tursi (38) also found that 87% of CD patients had normalized orocaecal transit time after the treatment with rifaximin. In conclusion, antibiotic treatment may improve GI symptoms and normalize breath tests in SIBO-positive patients.

## Discussion

The development or progression of IBD was widely suggested to be significantly associated with gut microbiota (1, 44–46). The link between IBD and SIBO has been previously presented in two meta-analyses (8, 9). Since then, increasing studies have been implemented to clarify the relationship between SIBO and IBD further. We exhaustively summarized the relevant data from 29 studies to conduct the updated meta-analysis. The included studies were conducted in 14 countries around the world. The sample size of our meta-analysis is about triple that of the previous meta-analysis (8).

Dysbiosis is one of the main pathogenesis of IBD (1, 45). The gut microbiota of a healthy population is mainly composed of the phyla Firmicutes, Bacteroidetes, Actinobacteria, and Verrucomicrobia (47). The dysbiosis in IBD patients is mainly characterized by a reduction of Firmicutes and Bacteroidetes and a relative increase of Proteobacteria (48). The gut microbiota produces local neurotransmitters, biologically active catecholamines, and metabolites and influences the gut-brain axis, thus playing an essential role in the pathogenesis of IBD (49–51). In our study, the pooled prevalence of SIBO among IBD patients was 31.0%, ranging from 8.9 to 61.9%. The odds of SIBO were 5.25-fold higher in IBD patients as compared to healthy controls. These conclusions are inconsistent with the previous studies (8, 9). Subgroup analysis showed that the odds of SIBO were higher in patients with CD (32.2%) compared to those with UC (27.8%), but the odds of SIBO were increased in both UC (OR = 4.22) and CD patients (OR = 7.34) compared to healthy controls. One possible reason is that the impaired ileocaecal valve in CD patients cannot prevent retrograde translocation of colonic bacteria. Thus, SIBO occurs more easily (52, 53). Differences in SIBO diagnostic tests may account for the variance of reported SIBO prevalence among IBD. In our study, the prevalence of SIBO diagnosed by LBT was prominently higher than that of GBT (43.3% vs. 22.1%). Glucose is a monosaccharide rapidly absorbable in the proximal small bowel, resulting in a low sensitivity for diagnosing SIBO (54). In contrast, lactulose is a disaccharide delivered early to colonic bacteria, producing excess hydrogen gas and a higher false-positive result (55). In addition, SIBO is more common among IBD patients in Eastern countries than in Western countries (34.7% vs. 28.4%). This might be a result of the differences in diets and metabolism among different geographic areas. In Eastern countries, the mainstream diet is carbohydrates (starch, sugars), which would increase the relative abundance of Bifidobacteria (56). Finally, the prevalence of SIBO among IBD patients from high-quality studies was higher than those from moderate-quality studies. Although uncertain, a greater number of studies using LBT in the high-quality group may explain the high prevalence of SIBO.

Gastrointestinal symptoms seen in IBD may overlap with those associated with SIBO, such as abdominal pain, diarrhea, and flatulence. According to previous studies (25, 33), antibiotic treatment can effectively improve symptoms of IBD among those with SIBO by altering the gut microbiota. Given the high prevalence of SIBO in individuals with IBD, identifying predictors of SIBO is important for IBD patients to achieve the maximum possible gain from antibiotic treatment. In our meta-analysis, IBD patients with SIBO had a lower BMI as compared to those without SIBO (MD = −1.04, *p* = 0.01). A reasonable explanation is that bacteria and/or their metabolites in SIBO will damage the epithelial barrier, leading to Inflammatory response and enhanced intestinal permeability, ultimately resulting in significant weight loss and malnutrition (57, 58). The luminal competition with the host for nutrients in SIBO may further contribute to malnutrition (59). Furthermore, the risks of bloating (OR = 3.02, *p* = 0.02) and flatulence (OR = 4.70, *p* = 0.01) were increased in SIBO-positive IBD patients. Bloating and flatulence were typical symptoms of SIBO, which may be associated with excessive production of hydrogen and intestinal motility disorders (5). Lastly, SIBO was positively associated with a history of abdominal surgery (OR = 2.05, *p* = 0.0007) and stricturing/penetrating disease (OR = 3.51, *p* = 0.0009) in IBD patients. Altered GI anatomy damages the integrality of the ileocecal valve and antegrade motility of the ileum, leading to retrograde translocation of colonic bacteria, which may predispose them to SIBO (6, 60).

Strengths of our study include an exhaustive literature search, careful analysis of variability of SIBO prevalence, and exploration of predictors of SIBO in IBD. There are limitations to our research. None of the included studies used jejunal aspirate and culture, the gold standard to diagnose SIBO. Copious definitions of positive breath tests in the included studies may contribute to heterogeneity in estimating SIBO prevalence in IBD. In addition, the asymmetry of the funnel plot calculating the pooled prevalence of SIBO suggested that the prevalence of SIBO in IBD may have been overestimated.

In summary, our meta-analysis has shown that nearly one-third of individuals with IBD present with SIBO positive, and the prevalence of SIBO varied according to the SIBO diagnostic methods performed. The odds of SIBO in IBD was increased by 5.25-fold compared with healthy individuals. Lower BMI, bloating, flatulence, history of abdominal surgery, and stricturing/penetrating disease behavior were predictors of SIBO in IBD.

## Data Availability

The original contributions presented in the study are included in the article/Supplementary material, further inquiries can be directed to the corresponding author.
