# Supplementary material for: Prevalence and predictors of small intestinal bacterial overgrowth in inflammatory bowel disease: a meta-analysis
Source: Front Med (Lausanne). 2025 Jan 21;11:1490506. doi: 10.3389/fmed.2024.1490506 (PMC11792544; doi:10.3389/fmed.2024.1490506)
Supplement: Supplementary file 1 [file Table_1.DOCX]

**Supplementary Material for manuscript:** Prevalence and predictors of small intestinal bacterial overgrowth in inflammatory bowel disease: A meta-analysis

**Legend of Figures:**

**Fig. S1:** Forest plot of studies showing the prevalence of SIBO based on IBD subtypes (32.2% vs 27.8%)

**Fig. S2:** Forest plot of studies showing the prevalence of SIBO based on SIBO diagnostic tests (43.3% vs 22.1% vs 22.7%)

**Fig. S3:** Forest plot of studies showing the prevalence of SIBO based on quality of study (32.2% vs 27.8%)

**Fig. S4:** Forest plot of studies showing the prevalence of SIBO based on geographic areas (34.7% vs 28.4%)

**Fig. S5:** Forest plot of odds ratios of SIBO in UC patients compared with healthy controls (OR=4.22; 95%CI 2.26 -7.85), (I^2^=45.5, p=0.076)

**Fig. S6:** Forest plot of odds ratios of SIBO in CD patients compared with healthy controls (OR=7.34; 95%CI 2.69 - 20.05), (I^2^=73.0, p=0.001)

**Fig. S7:** Forest plot of MBI in IBD patients with SIBO compared to those without SIBO (mean difference (MD) = -1.04; 95% CI -1.86- -0.23; p=0.01), (I^2^=0, p=0.52)

**Fig. S8:** Forest plot of odds ratios of bloating in IBD patients with SIBO compared to those without SIBO (OR=3.02; 95% CI 1.22-7.51; p=0.02), (I^2^=86, p＜0.00001)

**Fig. S9:** Forest plot of odds ratios of flatulence in IBD patients with SIBO compared to those without SIBO (OR=4.70; 95% CI 1.44-15.35; p=0.01), (I^2^=89, p＜0.00001)

**Fig. S10:** Forest plot of odds ratios of history of abdominal surgery in IBD patients with SIBO compared to those without SIBO (OR=2.05; 95% CI 1.35-3.11; p=0.0007), (I^2^=63, p=0.0008)

**Fig. S11:** Forest plot of odds ratios of CD behavior in IBD patients with SIBO compared to those without SIBO (OR=3.51; 95% CI 1.67-7.40; p=0.0009), (I^2^=84, p＜0.00001)

**Fig. S12:** Forest plot of mean age in IBD patients with SIBO compared to those without SIBO (MD=0.90; 95% CI -1.88 – 3.69; p=0.53), (I^2^=29, p=0.22)

**Fig. S13:** Forest plot of odds ratios of male in IBD patients with SIBO compared to those without SIBO (OR=0.82; 95% CI 0.57 – 1.18; p=0.29), (I^2^=27, p=0.21)

**Fig. S14:** Forest plot of disease duration in IBD patients with SIBO compared to those without SIBO (MD=0.40; 95% CI -0.08 – 0.88; p=0.10), (I^2^=0, p=0.42)

**Fig. S15:** Forest plot of odds ratios of disease location in IBD patients with SIBO compared to those without SIBO (OR=1.06; 95% CI 0.79 – 1.43; p=0.69), (I^2^=0, p=0.77)

**Fig. S16:** Forest plot of odds ratios of abdominal pain in IBD patients with SIBO compared to those without SIBO (OR=1.48; 95% CI 0.97 – 2.27; p=0.07), (I^2^=42, p=0.10)

**Fig. S17:** Forest plot of odds ratios of diarrhea in IBD patients with SIBO compared to those without SIBO (OR=1.33; 95% CI 0.70 – 2.50; p=0.38), (I^2^=62, p=0.03)

**Fig. S18:** Forest plot of odds ratios of steroids in IBD patients with SIBO compared to those without SIBO (OR=0.75; 95% CI 0.44 – 1.28; p=0.30), (I^2^=0, p=0.62)

**Fig. S19:** Forest plot of odds ratios of immunomodulator in IBD patients with SIBO compared to those without SIBO (OR=1.10; 95% CI 0.73 – 1.65; p=0.65), (I^2^=0, p=0.88)

**Fig. S20:** Forest plot of odds ratios of smoking in IBD patients with SIBO compared to those without SIBO (OR=1.23; 95% CI 0.77 – 1.96; p=0.38), (I^2^=0, p=0.99)

**Legend of Tables:**

**Table S1:** Joanna Briggs Institute (JBI) Critical Appraisal Tools for quality assessment of case-series study assessing the prevalence of SIBO in IBD patients included in the meta-analysis.

**Table S2:** Newcastle-Ottawa scale for assessment of quality of case-control studies assessing the prevalence of SIBO in IBD patients included in the meta-analysis.

**Table S3:** Assessment of risk factors and cut off criteria for diagnosing SIBO in IBD patients in the meta-analysis.

**Table S4:** Studies evaluating the effect of antibiotic treatment in IBD patients with SIBO.


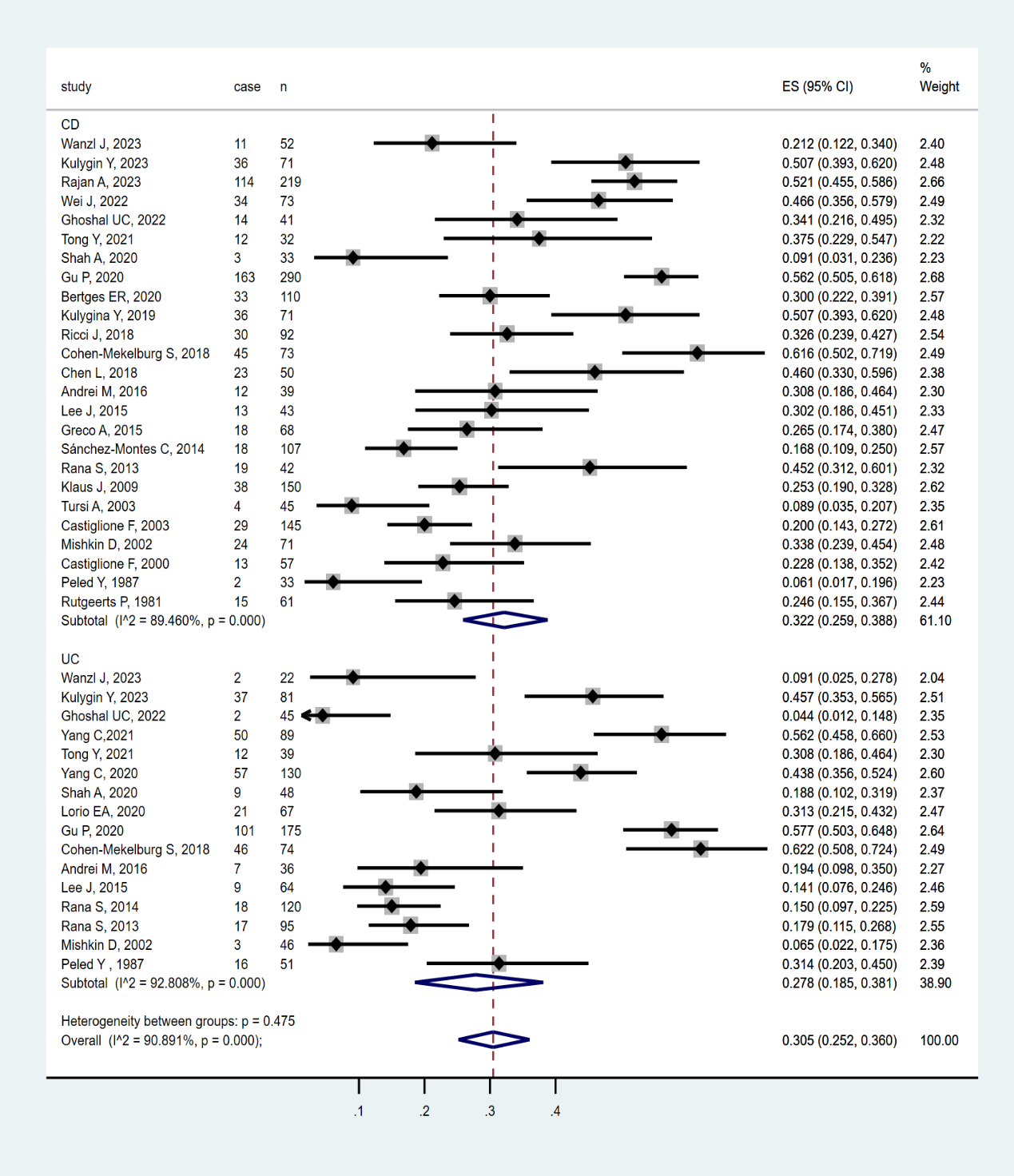


**Fig. S1:** Forest plot of studies showing the prevalence of SIBO based on IBD subtypes (32.2% vs 27.8%)


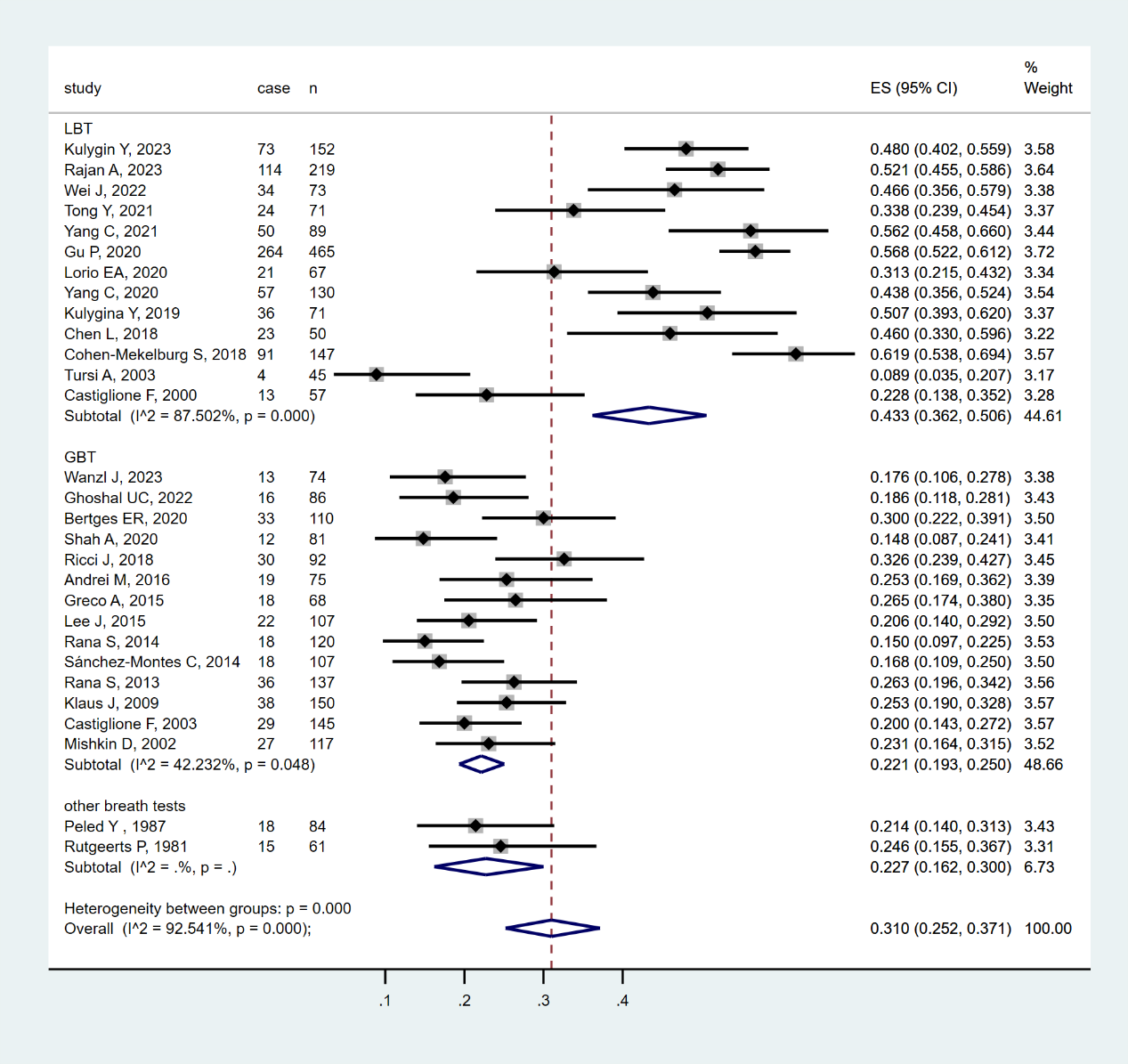


**Fig. S2:** Forest plot of studies showing the prevalence of SIBO based on SIBO diagnostic tests (43.3% vs 22.1% vs 22.7%)


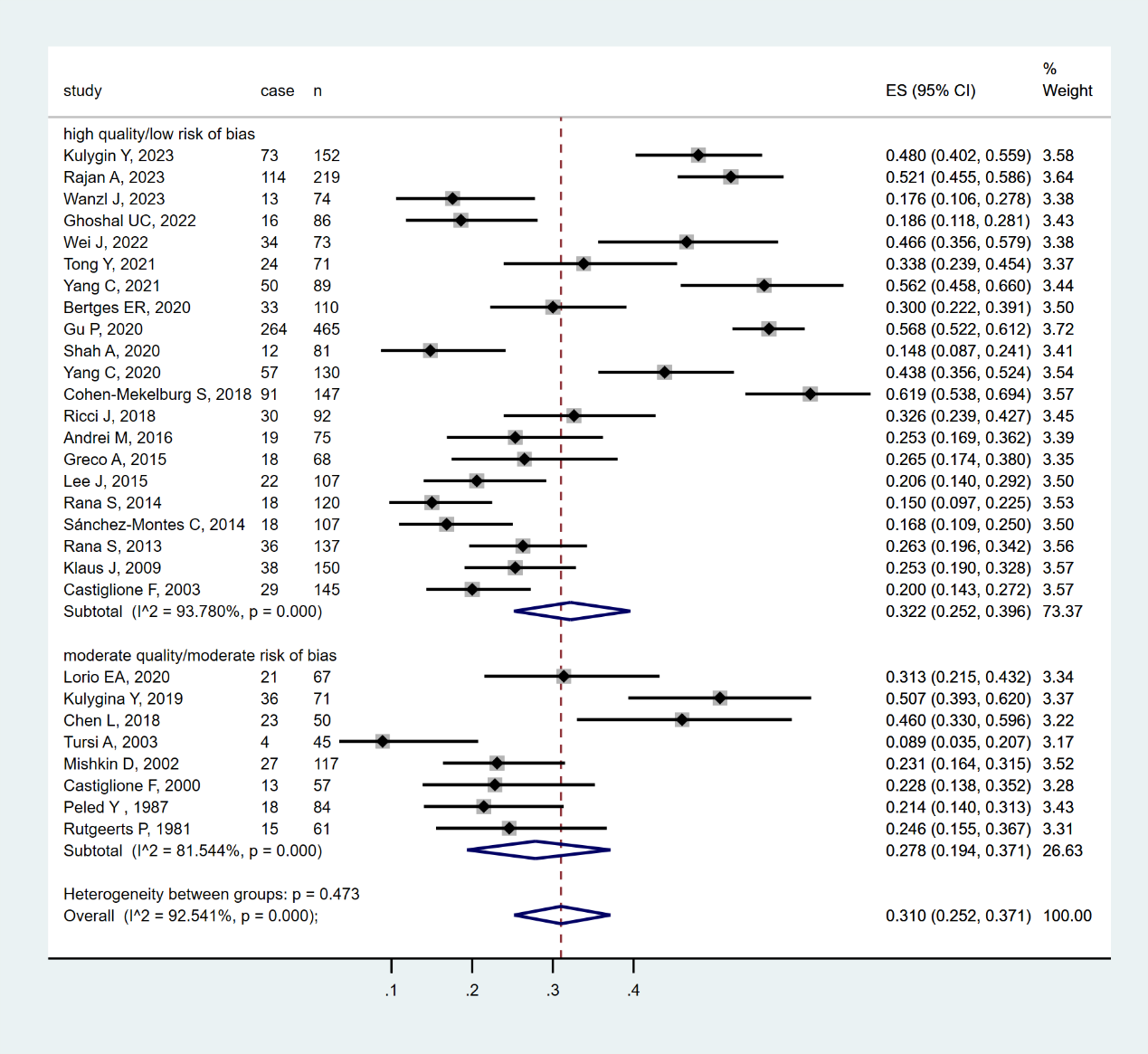


**Fig. S3:** Forest plot of studies showing the prevalence of SIBO based on quality of study (32.2% vs 27.8%)


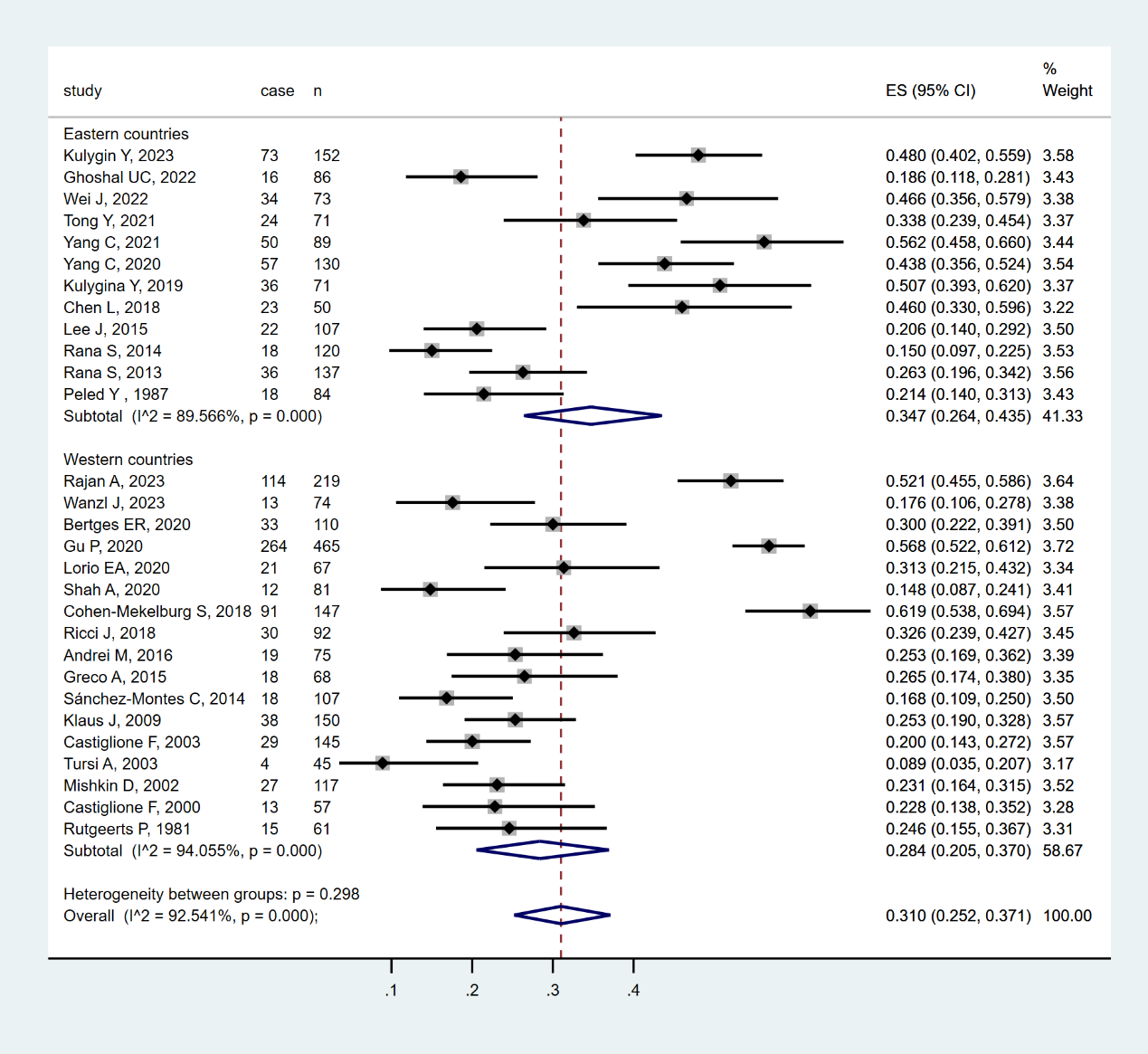


**Fig. S4:** Forest plot of studies showing the prevalence of SIBO based on geographic areas (34.7% vs 28.4%)


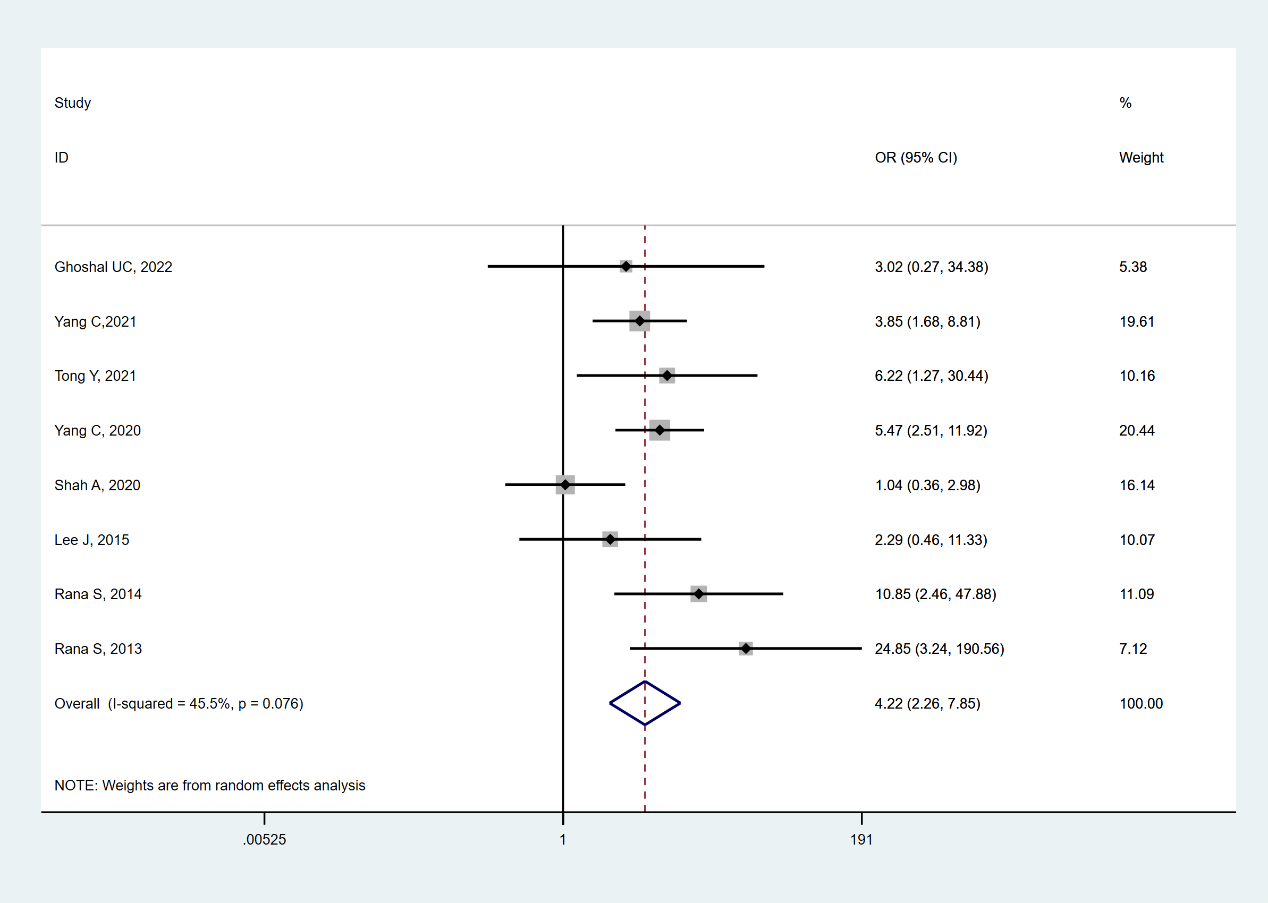


**Fig. S5:** Forest plot of odds ratios of SIBO in UC patients compared with healthy controls (OR=4.22; 95%CI 2.26 -7.85), (I^2^=45.5, p=0.076)


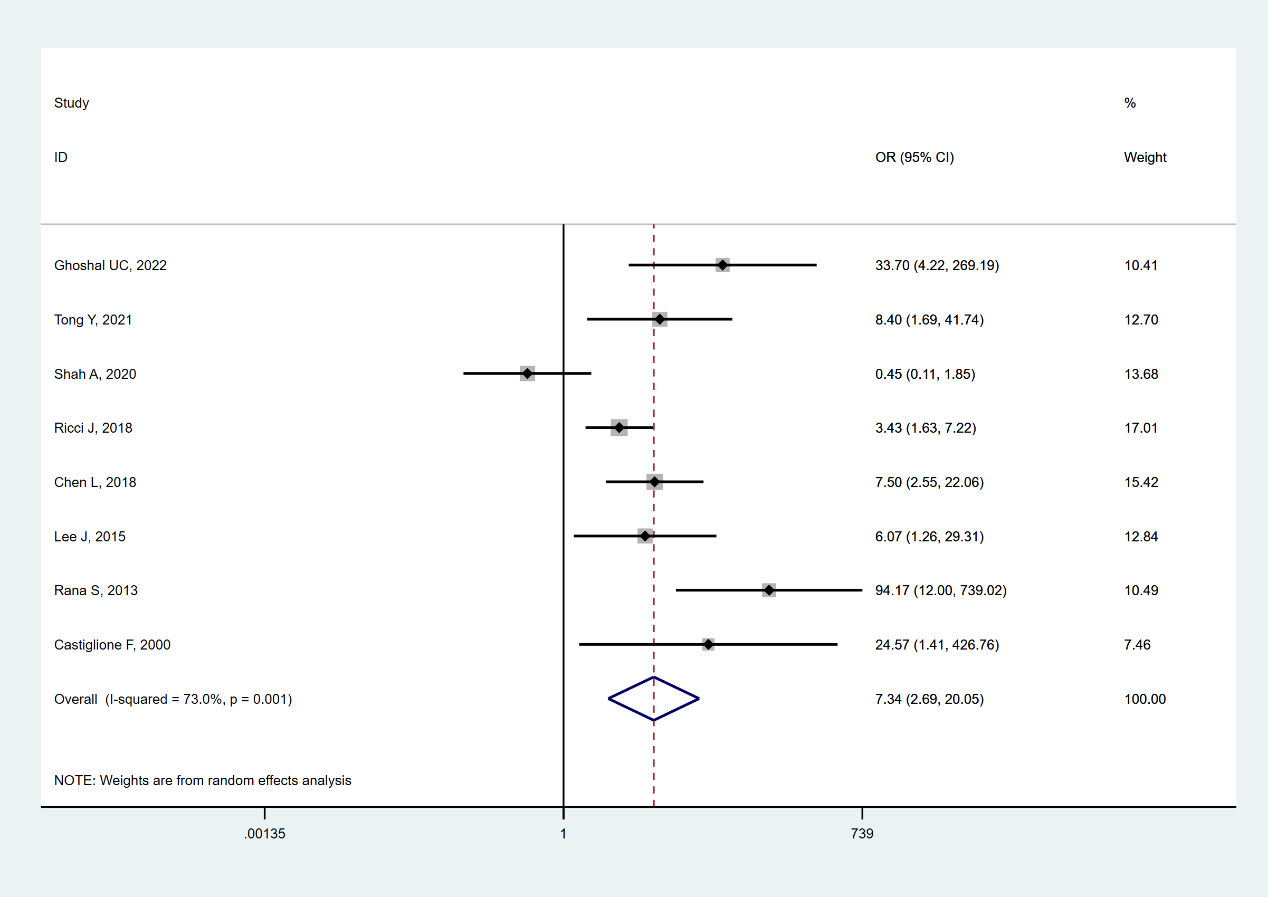


**Fig. S6:** Forest plot of odds ratios of SIBO in CD patients compared with healthy controls (OR=7.34; 95%CI 2.69 - 20.05), (I^2^=73.0, p=0.001)


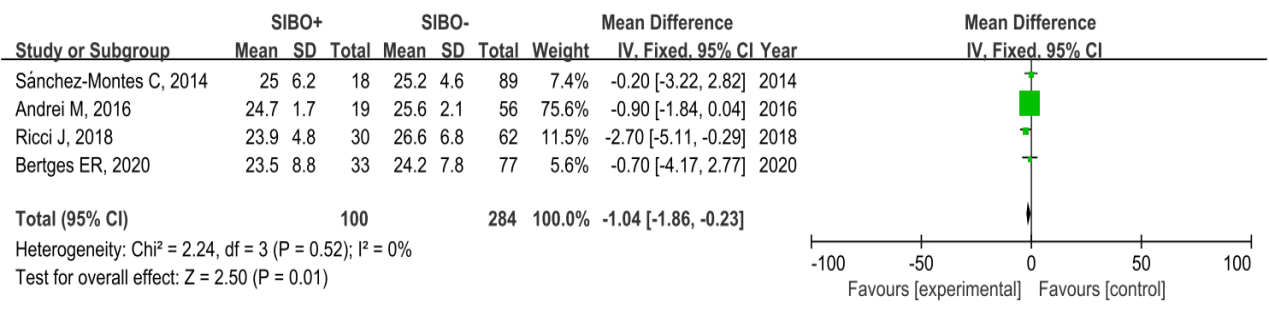


**Fig. S7:** Forest plot of MBI in IBD patients with SIBO compared to those without SIBO (mean difference (MD) = -1.04; 95% CI -1.86- -0.23; p=0.01), (I^2^=0, p=0.52)


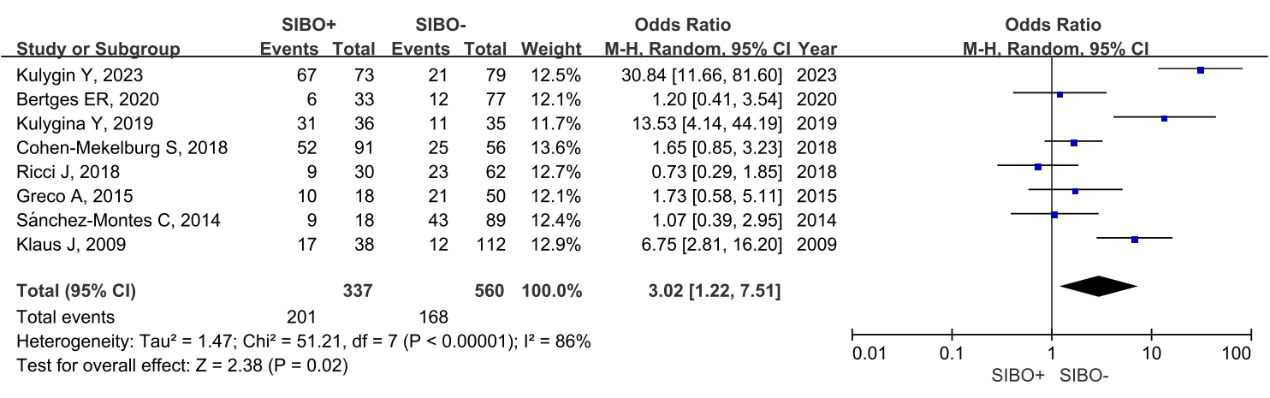


**Fig. S8:** Forest plot of odds ratios of bloating in IBD patients with SIBO compared to those without SIBO (OR=3.02; 95% CI 1.22-7.51; p=0.02), (I^2^=86, p＜0.00001)


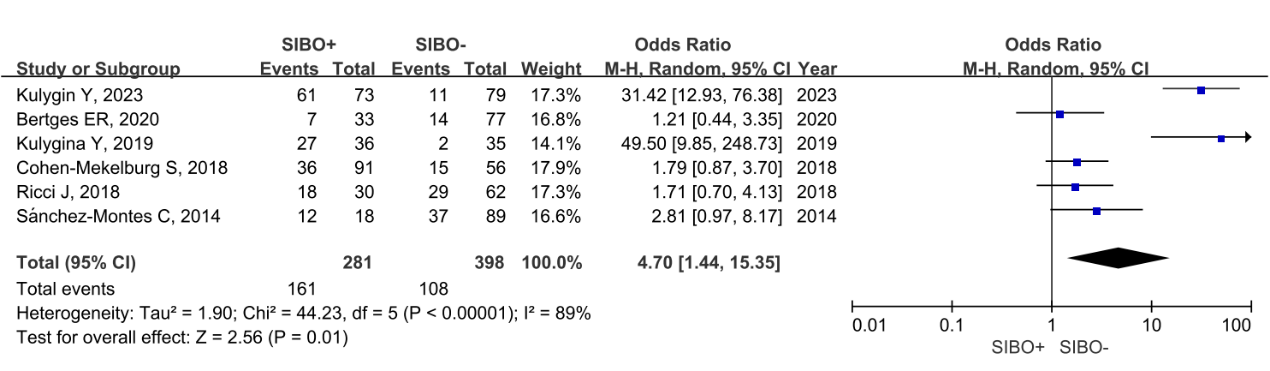


**Fig. S9:** Forest plot of odds ratios of flatulence in IBD patients with SIBO compared to those without SIBO (OR=4.70; 95% CI 1.44-15.35; p=0.01), (I^2^=89, p＜0.00001)


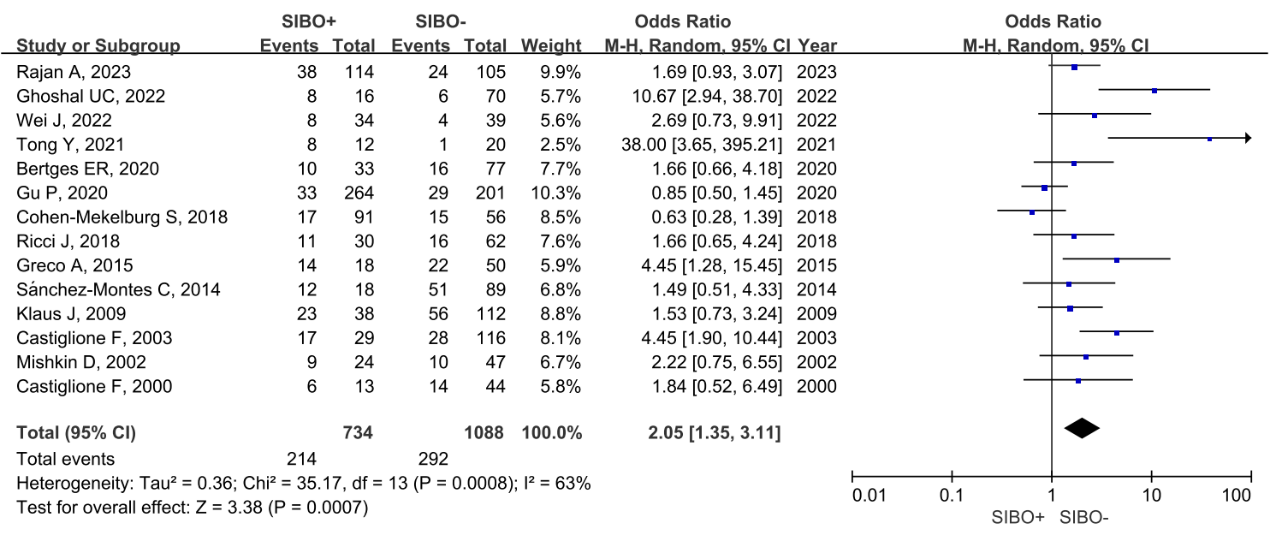


**Fig. S10:** Forest plot of odds ratios of history of abdominal surgery in IBD patients with SIBO compared to those without SIBO (OR=2.05; 95% CI 1.35-3.11; p=0.0007), (I^2^=63, p=0.0008)


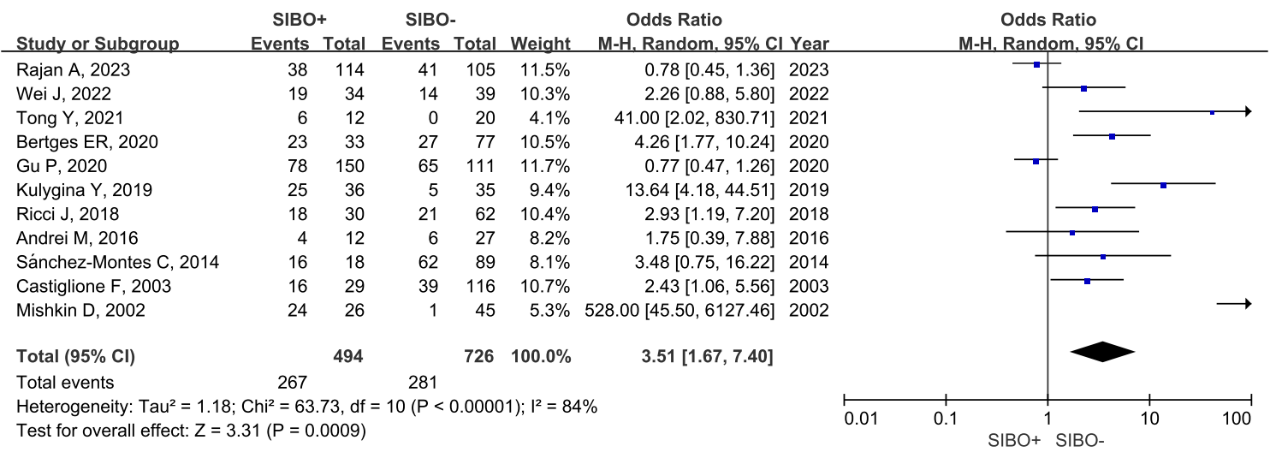


**Fig. S11:** Forest plot of odds ratios of CD behavior in IBD patients with SIBO compared to those without SIBO (OR=3.51; 95% CI 1.67-7.40; p=0.0009), (I^2^=84, p＜0.00001)


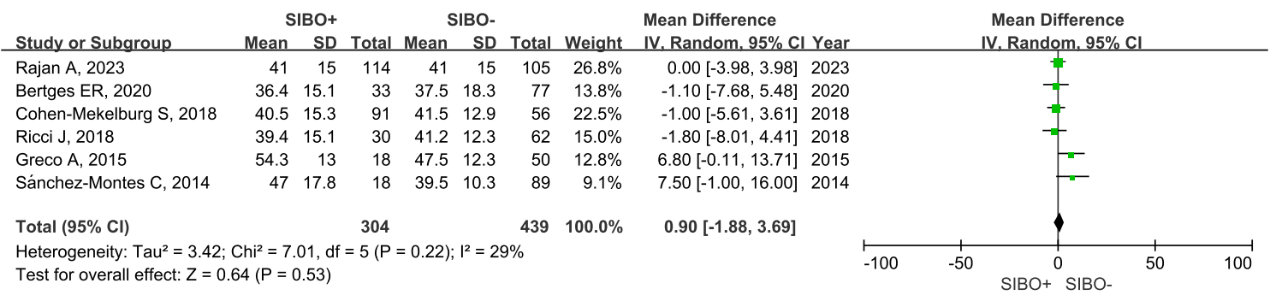


**Fig. S12:** Forest plot of mean age in IBD patients with SIBO compared to those without SIBO (MD=0.90; 95% CI -1.88 – 3.69; p=0.53), (I^2^=29, p=0.22)


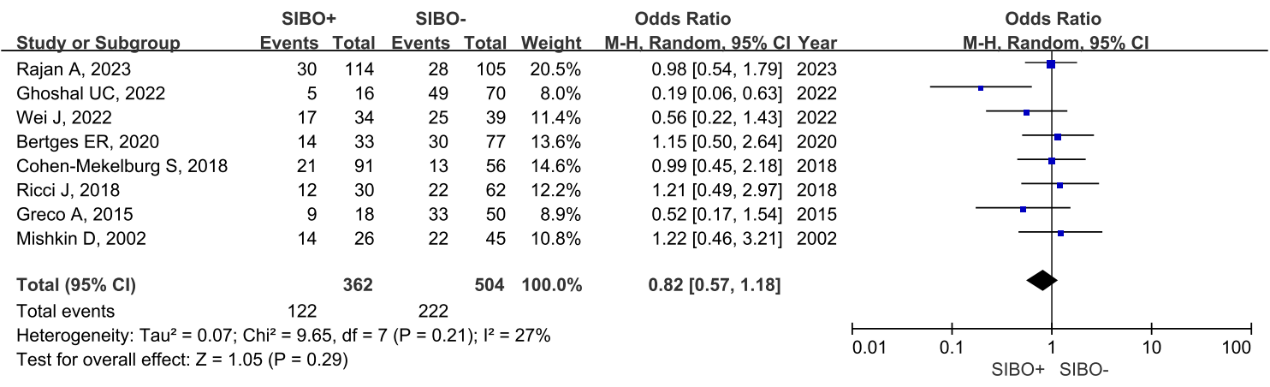


**Fig. S13:** Forest plot of odds ratios of male in IBD patients with SIBO compared to those without SIBO (OR=0.82; 95% CI 0.57 – 1.18; p=0.29), (I^2^=27, p=0.21)


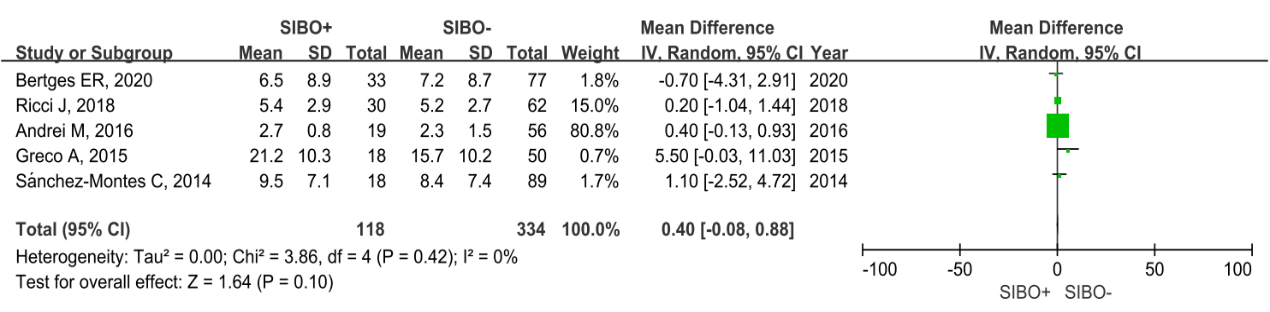


**Fig. S14:** Forest plot of disease duration in IBD patients with SIBO compared to those without SIBO (MD=0.40; 95% CI -0.08 – 0.88; p=0.10), (I^2^=0, p=0.42)


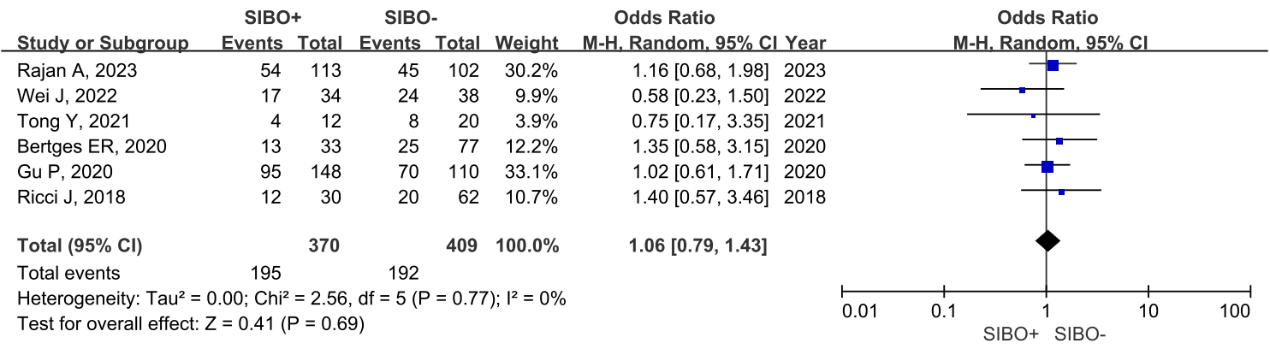


**Fig. S15:** Forest plot of odds ratios of disease location in IBD patients with SIBO compared to those without SIBO (OR=1.06; 95% CI 0.79 – 1.43; p=0.69), (I^2^=0, p=0.77)


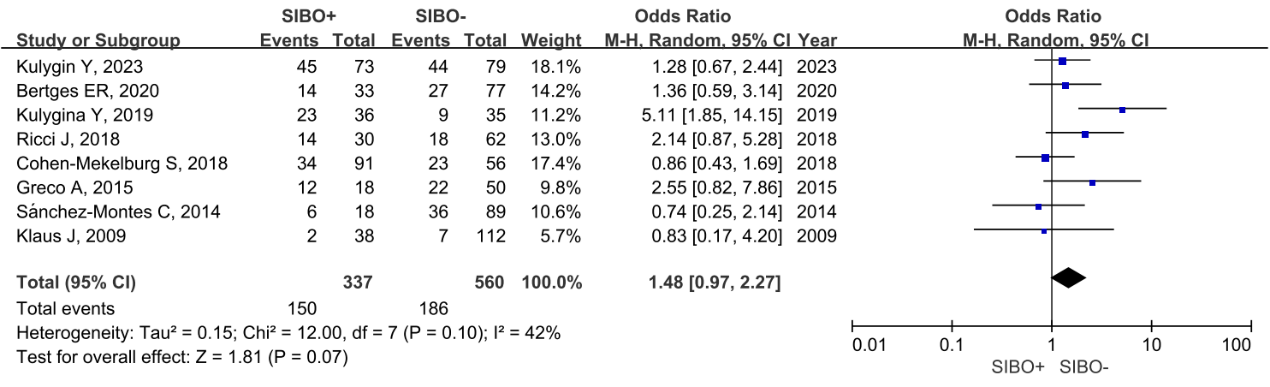


**Fig. S16:** Forest plot of odds ratios of abdominal pain in IBD patients with SIBO compared to those without SIBO (OR=1.48; 95% CI 0.97 – 2.27; p=0.07), (I^2^=42, p=0.10)


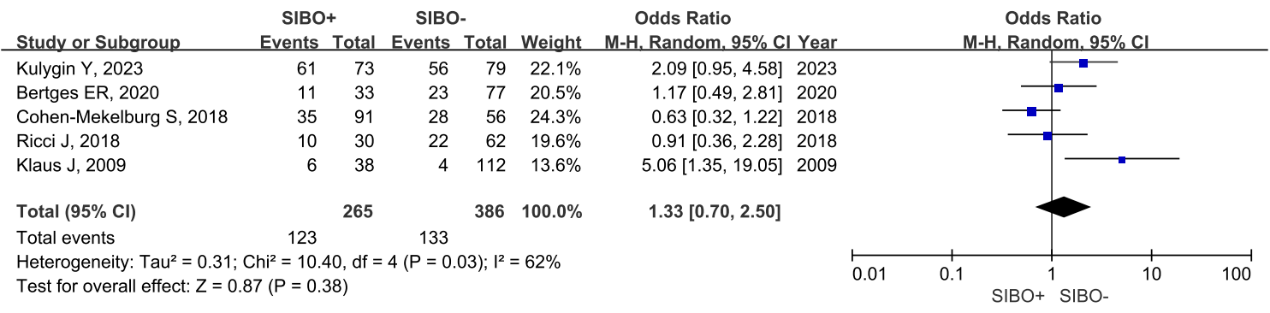


**Fig. S17:** Forest plot of odds ratios of diarrhea in IBD patients with SIBO compared to those without SIBO (OR=1.33; 95% CI 0.70 – 2.50; p=0.38), (I^2^=62, p=0.03)


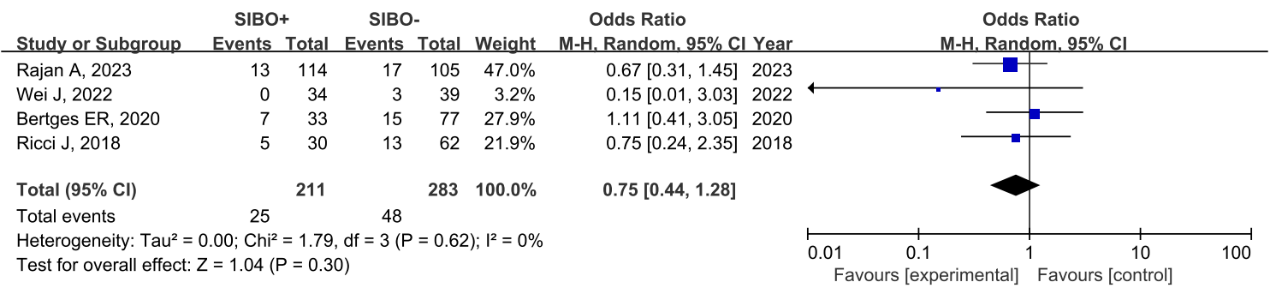


**Fig. S18:** Forest plot of odds ratios of steroids in IBD patients with SIBO compared to those without SIBO (OR=0.75; 95% CI 0.44 – 1.28; p=0.30), (I^2^=0, p=0.62)


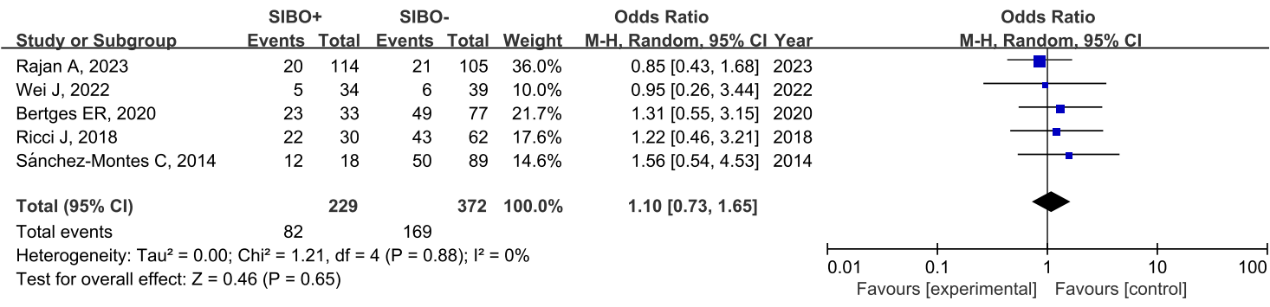


**Fig. S19:** Forest plot of odds ratios of immunomodulator in IBD patients with SIBO compared to those without SIBO (OR=1.10; 95% CI 0.73 – 1.65; p=0.65), (I^2^=0, p=0.88)


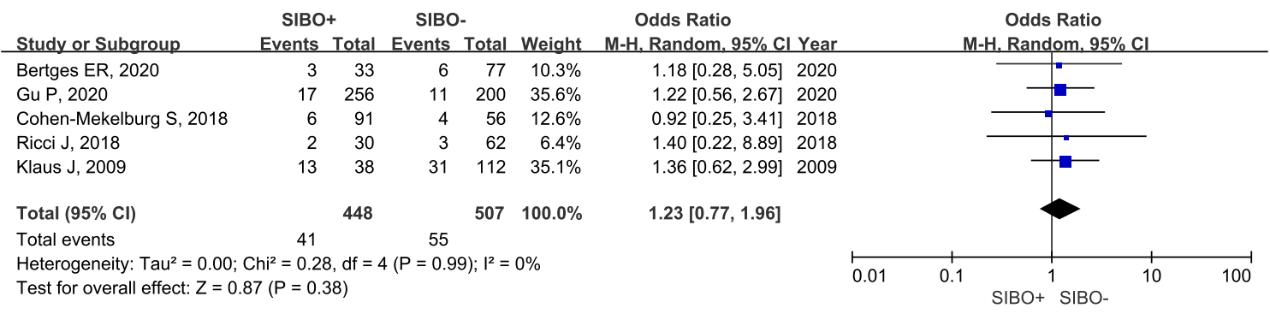


**Fig. S20:** Forest plot of odds ratios of smoking in IBD patients with SIBO compared to those without SIBO (OR=1.23; 95% CI 0.77 – 1.96; p=0.38), (I^2^=0, p=0.99)

**Table S1:** Joanna Briggs Institute (JBI) Critical Appraisal Tools for quality assessment of case-series study assessing the prevalence of SIBO in IBD patients included in the meta-analysis.

| No | Author | 1. Was the sample frame appropriate to address the target population? | 2. Were study participants sampled in an appropriate way? | 3. Was the sample size adequate? | 4. Were the study subjects and the setting described in detail? | 5. Was the data analysis conducted with sufficient coverage of the identified sample? | 6. Were valid methods used for the identification of the condition? | 7. Was the condition measured in a standard, reliable way for all participants? | 8. Was there appropriate statistical analysis? | 9. Was the response rate adequate, and if not, was the low response rate managed appropriately? | Risk of bias |
| --- | --- | --- | --- | --- | --- | --- | --- | --- | --- | --- | --- |
| 1 | Wanzl J ^[1]^ | Y | Y | Y | Y | Y | unclear | Y | Y | Y | low |
| 2 | Kulygin Y ^[2]^ | Y | unclear | Y | N | Y | Y | Y | Y | Y | low |
| 3 | Rajan A ^[3]^ | Y | unclear | Y | Y | Y | Y | Y | Y | Y | low |
| 4 | Wei J ^[4]^ | Y | Y | Y | Y | Y | Y | Y | Y | Y | low |
| 5 | Lorio EA ^[10]^ | Y | Y | Y | N | Y | Y | N | Y | N | moderate |
| 6 | Gu, P ^[11]^ | Y | N | Y | Y | Y | Y | Y | Y | Y | low |
| 7 | Bertges ER ^[12]^ | Y | Y | Y | Y | Y | Y | Y | Y | Y | low |
| 8 | Kulygina Y ^[13]^ | Y | unclear | Y | N | Y | Y | N | Y | Y | moderate |
| 9 | Cohen-Mekelburg, S ^[15]^ | Y | Y | Y | Y | Y | Y | Y | Y | Y | low |
| 10 | Andrei, M ^[17]^ | Y | Y | Y | Y | Y | Y | Y | Y | Y | low |
| 11 | Greco, A ^[19]^ | Y | Y | Y | Y | Y | Y | Y | Y | Y | low |
| 12 | Sánchez-Montes, C ^[20]^ | Y | Y | Y | Y | Y | Y | Y | Y | Y | low |
| 13 | Klaus, J ^[23]^ | Y | Y | Y | Y | Y | Y | Y | Y | Y | low |
| 14 | Tursi, A ^[24]^ | Y | Y | N | Y | N | N | Y | Y | Y | moderate |
| 15 | Castiglione, F ^[25]^ | Y | unclear | Y | Y | Y | Y | Y | Y | Y | low |
| 16 | Mishkin, D ^[26]^ | Y | unclear | Y | N | Y | N | Y | Y | Y | moderate |
| 17 | Peled Y ^[28]^ | Y | unclear | Y | N | Y | N | Y | Y | Y | moderate |
| 18 | Rutgeerts, P ^[29]^ | Y | unclear | Y | N | Y | N | Y | Y | Y | moderate |

Y: yes; N: no

**Table S2:** Newcastle-Ottawa scale for assessment of quality of case-control studies assessing the prevalence of SIBO in IBD patients included in the meta-analysis.

| **No** | **Author** | **Selection** | | | | **Comparability** | | **Exposure** | | |  |
| --- | --- | --- | --- | --- | --- | --- | --- | --- | --- | --- | --- |
|  |  | Is the case definition adequate? | Representativeness of the cases | Selection of Controls | Definition of Controls | Study controls for single factor | Study controls for additional factors | Ascertainment of exposure | Same method of ascertainment for cases and controls | Non-Response rate | Overall Quality Score  (Maximum = 9) |
| 1 | Ghoshal UC ^[5]^ | * | * | * | * | * | * | * | * | * | 9 |
| 2 | Yang C ^[6]^ | * | * | - | * | * | * | * | * | * | 8 |
| 3 | Tong Y ^[7]^ | * | * | * | * | * | * | * | * | * | 9 |
| 4 | Yang C ^[8]^ | * | * | - | * | * | * | * | * | * | 8 |
| 5 | Shah A ^[9]^ | * | * | * | * | * | * | * | * | - | 8 |
| 6 | Ricci, J ^[14]^ | * | * | * | * | * | * | * | * | * | 9 |
| 7 | Chen, L ^[16]^ | * | * | - | * | - | - | * | * | * | 6 |
| 8 | Lee, J ^[18]^ | * | * | * | * | * | * | * | * | * | 9 |
| 9 | Rana, S ^[21]^ | * | * | * | * | * | * | * | * | * | 9 |
| 10 | Rana, S ^[22]^ | * | - | - | * | * | * | * | * | * | 7 |
| 11 | Castiglione, F ^[27]^ | * | - | - | * | * | * | * | * | - | 6 |

* Each asterisk represents if individual criterion within the subsection was fulfilled.

**Table S3:** Assessment of risk factors and cut off criteria for diagnosing SIBO in IBD patients in the meta-analysis.

| **No** | **Author** | **Diagnostic test of SIBO** | **Dose of substrate** | **Cut off criteria for positive SIBO diagnosis** | **Prior antibiotic use** | **Concurrent PPI use** | **Prior Surgery** |
| --- | --- | --- | --- | --- | --- | --- | --- |
| 1 | Wanzl J ^[1]^ | GBT | 75g glucose | a rise of hydrogen and/or methane gas > 20 ppm above baseline | NO | NO | NA |
| 2 | Kulygin Y ^[2]^ | LBT | 10g lactulose | a rise of hydrogen gas >20 ppm above baseline within 90 minutes | NO | NA | NO |
| 3 | Rajan A ^[3]^ | LBT | 10g lactulose | ① a rise of hydrogen gas >20 ppm above baseline within 90 minutes;  ② a rise of methane gas >10 ppm at any point during the test. | NO | NA | 61 |
| 4 | Wei J ^[4]^ | LBT | 10g lactulose | ① a rise of hydrogen gas >20 ppm;  ② a rise of methane gas >12 ppm;  ③a rise of hydrogen and/or methane gas > 15 ppm above baseline | NA | NA | 12 |
| 5 | Ghoshal UC ^[5]^ | GBT | 100g glucose | a rise of hydrogen gas >12 ppm above baseline | NA | NA | 13 |
| 6 | Yang C ^[6]^ | LBT | 10g lactulose | ① hydrogen concentration > 20 ppm;  ② a rise of methane gas >12 ppm;  ③ a rise of combined hydrogen and methane gas >15ppm; | NO | NO | NO |
| 7 | Tong Y ^[7]^ | LBT | 10g lactulose | ① hydrogen concentration > 20 ppm;  ② a rise of hydrogen gas >12 ppm above baseline;  ③ a rise of methane gas >10 ppm above baseline | NO | NO | NA |
| 8 | Yang C ^[8]^ | LBT | unclear | ① hydrogen concentration > 20 ppm;  ② a rise of hydrogen gas >10 ppm; | NO | NA | NA |
| 9 | Shah A ^[9]^ | GBT | 75g glucose | ① a rise of hydrogen gas >20 ppm above baseline  ② a rise of methane gas >10 ppm above baseline | NO | 22 | NA |
| 10 | Lorio EA ^[10]^ | LBT | unclear | a rise of hydrogen gas >10 ppm | NA | NA | 9 |
| 11 | Gu P ^[11]^ | LBT | 10g lactulose | ① a rise of hydrogen gas >20 ppm;  ② a rise of methane gas >10 ppm; | NA | NA | 133 |
| 12 | Bertges ER ^[12]^ | GBT | 50g glucose | a rise of hydrogen and/or methane gas >12 ppm above baseline | NO | NO | NO |
| 13 | Kulygina Y ^[13]^ | LBT | unclear | unclear | NA | NA | NA |
| 14 | Ricci J ^[14]^ | GBT | 50g glucose | a rise of hydrogen and/or methane gas >12 ppm above baseline | NO | NO | NO |
| 15 | Cohen-Mekelburg S ^[15]^ | LBT | 10g lactulose | ① a rise of hydrogen gas > 20 ppm above baseline  ② a rise of methane gas > 12 ppm above baseline | NA | 36 | 32 |
| 16 | Chen L ^[16]^ | LBT | unclear | unclear | NA | NA | NA |
| 17 | Andrei M ^[17]^ | GBT | 50g glucose | a rise of hydrogen gas > 12 ppm above baseline | NO | NA | NA |
| 18 | Lee J ^[18]^ | GBT | 75g glucose | ① a rise of hydrogen gas > 12 ppm above baseline  ② a rise of methane gas >10 ppm above baseline | NO | NO | 13 |
| 19 | Greco A ^[19]^ | GBT | 75g glucose | a rise of hydrogen and/or methane gas > 12 ppm above baseline | NO | NA | 58 |
| 20 | Sánchez-Montes C ^[20]^ | GBT | 50g glucose | a rise of hydrogen gas > 12 ppm above baseline | NO | 19 | 63 |
| 21 | Rana S ^[21]^ | GBT | 80g glucose | a rise of hydrogen gas > 12 ppm above baseline | NO | NA | NO |
| 22 | Rana S ^[22]^ | GBT | 80g glucose | a rise of hydrogen and/or methane gas >12 ppm above baseline | NO | NA | NO |
| 23 | Klaus J ^[23]^ | GBT | 50g glucose | a rise of hydrogen gas > 10 ppm above baseline | NO | 13 | 23 |
| 24 | Tursi A ^[24]^ | LBT | 10g lactulose | hydrogen concentration > 20 ppm | NA | NA | NO |
| 25 | Castiglione F ^[25]^ | GBT | 50g glucose | a rise of hydrogen gas > 12 ppm above baseline | NO | NA | 45 |
| 26 | Mishkin D ^[26]^ | GBT | 50g glucose | a rise of hydrogen gas > 10 ppm above baseline | NO | NA | NA |
| 27 | Castiglione F ^[27]^ | LBT | 10g lactulose | a rise of hydrogen and/or methane gas > 12 ppm above baseline | NO | NA | 24 |
| 28 | Peled Y ^[28]^ | methane breath test | NA | Breath CH4 level was at least 1 ppm above ambient air | NA | NA | NO |
| 29 | Rutgeerts P ^[29]^ | 14C-glycocholate breath test | 5µ Ci14C-glycocholic acid | cumulative percentage of ^14^CO2 excretion in the breath 8 hours | NO | NA | NO |

LBT: lactulose breath test; GBT: glucose breath test; ppm: parts per million; NA: not applicable.

**Table S4:** Studies evaluating the effect of antibiotic treatment in IBD patients with SIBO.

| **No** | Author | Number of Patients | Treatment with antibiotic | Symptom improvement post treatment | Normalization of BT post treatment |
| --- | --- | --- | --- | --- | --- |
| 1 | Yang C ^[6]^ | Group A (n=22 UC patients with SIBO);  Group B (n=28 UC patients with SIBO) | Group A: mesalazine (1 g/time, 4 times/d, 6 weeks)  Group B: mesalazine (1 g/time, 4 times/d, 6 weeks) + rifaximin (0.2 g/time, 4 times/d, 2 weeks plus) | The group B presented greater total effective rate (symptom plus microscopic examination or Mayo score alone) compared with group A (92.86% vs. 63.64%, P<0.05).  The group B led to reduction in the level of ESR and CRP that was greater than group A (all P<0.05). | NA |
| 2 | Gu P ^[11]^ | 117 IBD patients with SIBO | Various antibiotics (2 weeks) | 57.3% patients achieved symptomatic improvement after antibiotics. | NA |
| 3 | Cohen-Mekelburg S ^[15]^ | 91 IBD patients with SIBO | 54 patients: combination of rifaximin and probiotics  21 patients: rifaximin alone  16 patients: probiotics alone | The median Mayo Score changed from 2.0 to 1.5 post treatment  the median HBI Score changed from 5.0 to 3.0 post treatment | NA |
| 4 | Greco A ^[19]^ | 15 CD patients with SIBO | 9 patients: ciprofloxacin (500 mg/day, 1 month);  4 patients: metronidazole (750 mg/day, 1 month);  2 patients: rifaximin (1200 mg/day, 1 month);  All patients received, at the end of antibiotic therapy, probiotics containing Lactobacillus casei DG for the following 2 months | Vitamin B12 levels after antibiotic and probiotic treatment increased significantly (249±147 pg/mL vs. 331±160 pg/mL, p=0.011, in pre-treatment and post-treatment, respectively). | 13/15 |
| 5 | Tursi A ^[24]^ | 45 CD patients with SIBO | rifaximin (800 mg/day, 7 days) | 87% patients had normalized orocaecal transit time | NA |
| 6 | Castiglione F ^[25]^ | group A (n=15 CD patients with SIBO);  group B (n=14 CD patients with SIBO) | Group A: metronidazole (250 mg, 3 times/d, 10 days);  Group B: ciprofloxacin (500 mg, 2 times/d, 10 days) | The scores of bloating, stool softness, abdominal pain significantly improved in the two groups post antibiotic treatment (all P<0.05). | Group A: 13/15  Group B: 14/14 |

BT: breath test; NA, not applicable

**REFERNCES for supplementary material:**

1. Wanzl J, Gröhl K, Kafel A, et al. Impact of Small Intestinal Bacterial Overgrowth in Patients with Inflammatory Bowel Disease and Other Gastrointestinal Disorders-A Retrospective Analysis in a Tertiary Single Center and Review of the Literature. *J Clin Med*. 2023;12(3):935. Published 2023 Jan 25. doi:10.3390/jcm12030935
2. Kulygin Y.U.A, Osipenko M.F. Excessive bacterial growth syndrome in small intestine burdens the clinical picture of inflammatory bowel diseases. Experimental and Clinical Gastroenterology. 2023 :4 (49-54). doi: 10.31146/1682-8658-ecg-212-4-49-54
3. Rajan A, Pan Y, Mahtani P, et al. The Impact of Confounders on Symptom-Endoscopic Discordances in Crohn's Disease. *Crohns Colitis 360*. 2023;5(2):otad017. Published 2023 Mar 28. doi:10.1093/crocol/otad017
4. Wei J, Feng J, Chen L, et al. Small intestinal bacterial overgrowth is associated with clinical relapse in patients with quiescent Crohn's disease: a retrospective cohort study. *Ann Transl Med*. 2022;10(14):784. doi:10.21037/atm-22-3335
5. Ghoshal UC, Yadav A, Fatima B, Agrahari AP, Misra A. Small intestinal bacterial overgrowth in patients with inflammatory bowel disease: A case-control study. *Indian J Gastroenterol*. 2022;41(1):96-103. doi:10.1007/s12664-021-01211-6
6. Yang C, Zhang X, Wang S, Huo X, Wang J. Small intestinal bacterial overgrowth and evaluation of intestinal barrier function in patients with ulcerative colitis. *Am J Transl Res*. 2021;13(6):6605-6610. Published 2021 Jun 15.
7. Tong Y, Yu X, Yu Y, et al. SMALL INTESTINAL BACTERIAL OVERGROWTH AND LOW-GRADE SYSTEMIC INFLAMMATION IN PATIENTS WITH INFLAMMATORY BOWEL DISEASE. Acta Medica Mediterranea. 2021 37:5 (2941-2946). doi: 10.19193/0393-6384_2021_5_454
8. Yang C, Guo X, Wang J, et al. Relationship between Small Intestinal Bacterial Overgrowth and Peripheral Blood ET, TLR2 and TLR4 in Ulcerative Colitis. *J Coll Physicians Surg Pak*. 2020;30(3):245-249. doi:10.29271/jcpsp.2020.03.245
9. Shah A, Talley NJ, Koloski N, et al. Duodenal bacterial load as determined by quantitative polymerase chain reaction in asymptomatic controls, functional gastrointestinal disorders and inflammatory bowel disease. *Aliment Pharmacol Ther*. 2020;52(1):155-167. doi:10.1111/apt.15786
10. Lorio E.A, Wisniewski N, Brown E.D, et al. An Analysis of Small Intestine Bacterial Overgrowth Rates in Ulcerative Colitis. American Journal of Gastroenterology 2020 115:SUPPL (S439-S440). doi: 10.14309/01.ajg.0000705456.97559.1b
11. Gu P, Patel D, Lakhoo K, et al. Breath Test Gas Patterns in Inflammatory Bowel Disease with Concomitant Irritable Bowel Syndrome-Like Symptoms: A Controlled Large-Scale Database Linkage Analysis. *Dig Dis Sci*. 2020;65(8):2388-2396. doi:10.1007/s10620-019-05967-y
12. Bertges ER, Chebli JMF. PREVALENCE AND FACTORS ASSOCIATED WITH SMALL INTESTINAL BACTERIAL OVERGROWTH IN PATIENTS WITH CROHN'S DISEASE: A RETROSPECTIVE STUDY AT A REFERRAL CENTER. Arq Gastroenterol. 2020;57(3):283-288. doi:10.1590/S0004-2803.202000000-64
13. Kulygina Y, Osipenko M, Skalinskaya M, et al. Small intestinal bacterial overgrowth in patients with Crohn's disease is not only associated with a more severe disease, but is also marked by dramatic changes in the gut microbiome. Journal of Crohn's and Colitis. 2019;13(s544):1876-4479. doi:10.1093/ecco-jcc/jjy222.965
14. Ricci JER Júnior, Chebli LA, Ribeiro TCDR, et al. Small-Intestinal Bacterial Overgrowth is Associated With Concurrent Intestinal Inflammation But Not With Systemic Inflammation in Crohn's Disease Patients. *J Clin Gastroenterol*. 2018;52(6):530-536. doi:10.1097/MCG.0000000000000803
15. Cohen-Mekelburg S, Tafesh Z, Coburn E, et al. Testing and Treating Small Intestinal Bacterial Overgrowth Reduces Symptoms in Patients with Inflammatory Bowel Disease. *Dig Dis Sci*. 2018;63(9):2439-2444. doi:10.1007/s10620-018-5109-1
16. Chen L. Y, Yuan B. S, Wei J, et al. Stenosis and endoscopical disease activity are independent risk factors for small intestinal bacterial overgrowth in Crohn's disease. Journal of Digestive Diseases. 2018;19(139):1751-2980. doi:10.1111/1751-2980.12665
17. Andrei M, Gologan S, Stoicescu A, Ionescu M, Nicolaie T, Diculescu M. Small Intestinal Bacterial Overgrowth Syndrome Prevalence in Romanian Patients with Inflammatory Bowel Disease. *Curr Health Sci J*. 2016;42(2):151-156. doi:10.12865/CHSJ.42.02.06
18. Lee JM, Lee KM, Chung YY, et al. Clinical significance of the glucose breath test in patients with inflammatory bowel disease. *J Gastroenterol Hepatol*. 2015;30(6):990-994. doi:10.1111/jgh.12908
19. Greco A, Caviglia GP, Brignolo P, et al. Glucose breath test and Crohn's disease: Diagnosis of small intestinal bacterial overgrowth and evaluation of therapeutic response. *Scand J Gastroenterol*. 2015;50(11):1376-1381. doi:10.3109/00365521.2015.1050691
20. Sánchez-Montes C, Ortiz V, Bastida G, et al. Small intestinal bacterial overgrowth in inactive Crohn's disease: influence of thiopurine and biological treatment. *World J Gastroenterol*. 2014;20(38):13999-14003. doi:10.3748/wjg.v20.i38.13999
21. Rana SV, Sharma S, Kaur J, et al. Relationship of cytokines, oxidative stress and GI motility with bacterial overgrowth in ulcerative colitis patients. *J Crohns Colitis*. 2014;8(8):859-865. doi:10.1016/j.crohns.2014.01.007
22. Rana SV, Sharma S, Malik A, et al. Small intestinal bacterial overgrowth and orocecal transit time in patients of inflammatory bowel disease. *Dig Dis Sci*. 2013;58(9):2594-2598. doi:10.1007/s10620-013-2694-x
23. Klaus J, Spaniol U, Adler G, Mason RA, Reinshagen M, von Tirpitz C C. Small intestinal bacterial overgrowth mimicking acute flare as a pitfall in patients with Crohn's Disease. *BMC Gastroenterol*. 2009;9:61. Published 2009 Jul 30. doi:10.1186/1471-230X-9-61
24. Tursi A, Brandimarte G, Giorgetti G, Nasi G. Assessment of orocaecal transit time in different localization of Crohn's disease and its possible influence on clinical response to therapy. *Eur J Gastroenterol Hepatol*. 2003;15(1):69-74. doi:10.1097/00042737-200301000-00012
25. Castiglione F, Rispo A, Di Girolamo E, et al. Antibiotic treatment of small bowel bacterial overgrowth in patients with Crohn's disease. *Aliment Pharmacol Ther*. 2003;18(11-12):1107-1112. doi:10.1046/j.1365-2036.2003.01800.x
26. Mishkin D, Boston FM, Blank D, Yalovsky M, Mishkin S. The glucose breath test: a diagnostic test for small bowel stricture(s) in Crohn's disease. *Dig Dis Sci*. 2002;47(3):489-494. doi:10.1023/a:1017991313789
27. Castiglione F, Del Vecchio Blanco G, Rispo A, et al. Orocecal transit time and bacterial overgrowth in patients with Crohn's disease. *J Clin Gastroenterol*. 2000;31(1):63-66. doi:10.1097/00004836-200007000-00015
28. Peled Y, Weinberg D, Hallak A, Gilat T. Factors affecting methane production in humans. Gastrointestinal diseases and alterations of colonic flora. *Dig Dis Sci*. 1987;32(3):267-271. doi:10.1007/BF01297052
29. Rutgeerts P, Ghoos Y, Vantrappen G, Eyssen H. Ileal dysfunction and bacterial overgrowth in patients with Crohn's disease. *Eur J Clin Invest*. 1981;11(3):199-206. doi:10.1111/j.1365-2362.1981.tb01841.x
